# The dissociative role of bursting and non-bursting neural activity in the oscillatory nature of functional brain networks

**DOI:** 10.1162/imag_a_00231

**Published:** 2024-07-17

**Authors:** Alix Cordier, Alison Mary, Marc Vander Ghinst, Serge Goldman, Xavier De Tiège, Vincent Wens

**Affiliations:** Université libre de Bruxelles (ULB), ULB Neuroscience Institute (UNI), Laboratoire de Neuroanatomie et Neuroimagerie translationnelles (LN ^2^ T), Brussels, Belgium; Université libre de Bruxelles (ULB), ULB Neuroscience Institute (UNI), Neuropsychology and Functional Neuroimaging Research Unit (UR2NF) at Centre de Recherches Cognition et Neurosciences (CRCN), Brussels, Belgium; Université libre de Bruxelles (ULB), Hôpital Universitaire de Bruxelles (HUB), CUB Hôpital Erasme, Department of Ear, Nose and Throat, and of Cervico-facial surgery, Brussels, Belgium; Université libre de Bruxelles (ULB), Hôpital Universitaire de Bruxelles (HUB), CUB Hôpital Erasme, Department of Nuclear Medicine, Brussels, Belgium; Université libre de Bruxelles (ULB), Hôpital Universitaire de Bruxelles (HUB), CUB Hôpital Erasme, Department of Translational Neuroimaging, Brussels, Belgium

**Keywords:** brain oscillations, magnetoencephalography, power bias, resting-state functional connectivity, transient bursts

## Abstract

The oscillatory nature of intrinsic brain networks is largely taken for granted in the systems neuroscience community. However, the hypothesis that brain rhythms—and by extension transient bursting oscillations—underlie functional networks has not been demonstrated*per se.*Electrophysiological measures of functional connectivity are indeed affected by the power bias, which may lead to artefactual observations of spectrally specific network couplings not genuinely driven by neural oscillations, bursting or not. We investigate this crucial question by introducing a unique combination of a rigorous mathematical analysis of the power bias in frequency-dependent amplitude connectivity with a neurobiologically informed model of cerebral background noise based on hidden Markov modeling of resting-state magnetoencephalography (MEG). We demonstrate that the power bias may be corrected by a suitable renormalization depending nonlinearly on the signal-to-noise ratio, with noise identified as non-bursting oscillations. Applying this correction preserves the spectral content of amplitude connectivity, definitely proving the importance of brain rhythms in intrinsic functional networks. Our demonstration highlights a dichotomy between spontaneous oscillatory bursts underlying network couplings and non-bursting oscillations acting as background noise but whose function remains unsettled.

## 1 Introduction

A central notion in human neuroscience is that brain function is organized into intrinsic functional networks (Engel et al., 2013) sustained by spontaneously interacting neural assemblies (Deco & Corbetta, 2011;Fox & Raichle, 2007). Large-scale brain networks were initially mapped using resting-state functional magnetic resonance imaging (fMRI) (Biswal et al., 1995;Fox et al., 2005). Their electrophysiology and relation to brain rhythms were then investigated using resting-state magnetoencephalography (MEG) (Brookes et al., 2011;de Pasquale et al., 2010;Hipp et al., 2012;Liu et al., 2010). Electrophysiological networks turned out to be spectrally specific (Brookes et al., 2014,^2016^;Tewarie et al., 2016;Wens, Mary, et al., 2014) and explainable in terms of sub-second transient “bursts” of brain rhythms (Baker et al., 2014;Coquelet et al., 2022;Hindriks & Tewarie, 2023;Seedat et al., 2020;Vidaurre, Hunt, et al., 2018). Further studies also revealed their metastable, supra-second-scale dynamics underlying cross-network binding (Wens et al., 2019) and versatile network topology (de Pasquale et al., 2012,^2016^;Della Penna et al., 2019) as well as their role in stimulus processing (Betti et al., 2018;Hawellek et al., 2013;Smith et al., 2009), task performance (O’Neill, Bauer, et al., 2015;O’Neill et al., 2017;Quinn et al., 2018), learning and memory (Higgins et al., 2021;Mary et al., 2017;Roshchupkina et al., 2022;Van Dyck et al., 2021b), and several brain disorders (Brookes et al., 2018;Naeije et al., 2019;Puttaert et al., 2020;Sitnikova et al., 2018;Sjøgård et al., 2020;Van Dyck et al., 2021a;Van Schependom et al., 2019).

The electrophysiology of intrinsic brain networks is strongly constrained by the amplitude envelope correlation (AEC) (O’Neill, Barratt, et al., 2015;Sadaghiani et al., 2022;Siegel et al., 2012). The AEC is a functional connectivity measure that focuses on the oscillation amplitude of neural assemblies, tracks the spontaneous rise and fall of transient brain rhythms (Hari & Salmelin, 1997;van Ede et al., 2018), and quantifies to what extent these rhythms tend to burst simultaneously in distinct brain areas (Baker et al., 2014;Hindriks & Tewarie, 2023;Seedat et al., 2020). The co-occurrence of transient oscillatory bursts is actually thought to underlie electrophysiological network connectivity. This is different from the “communication-through-coherence” phenomenon (Fries, 2005), which instead depends on the precise time lag between these oscillations as measured by phase-based connectivity (Siegel et al., 2012). Crucially, these two theories of large-scale neural binding assume from the start that functional networks reflect interacting brain oscillations; yet this foundational assumption may be challenged. Conventional wisdom holds that signal amplitude or power artificially modulates connectivity estimation (O’Neill, Bauer, et al., 2015;Muthukumaraswamy & Singh, 2011), an effect known as the*power bias*. The fact that electrophysiological functional networks are best delineated in theα(8–12 Hz) and theβ(12–30 Hz) frequency bands (Brookes et al., 2011;Hipp et al., 2012;Siems et al., 2016;Wens, Mary, et al., 2014), precisely where resting-state signals exhibit their highest power (Hari & Salmelin, 1997), thus legitimately raises doubts on the oscillatory nature of functional connectivity. In a nutshell, connectivity could appear smaller in the lower (δ,θ) and higher (γ) frequency bands not because it is genuinely smaller but because electrophysiological recordings are noisier in these bands, leading to connectivity underestimation. So the possibility remains that intrinsic functional connectivity is broadband rather than carried by specific brain rhythms—a hypothesis first raised byHipp and Siegel (2015). Experimental findings of broadband connectivity could lead to a paradigm shift in our conceptual understanding of the electrophysiology of functional networks (Cabral, Kringelbach, et al., 2014;Deco et al., 2011).

Here, we sought to prove or disprove the broadband connectivity hypothesis by directly disentangling the power bias from neurophysiological AEC in resting-state MEG recordings. We engineered a mathematically rigorous procedure that corrects for the power bias in spectrally resolved AEC. Critically, our procedure requires knowledge about the cerebral background processes that are not involved in amplitude coupling. This hinders*a priori*its applicability since un-mixing background and connectivity processes in resting-state data is a difficult, unsolved problem. We solved it here by incorporating a biologically informed model of amplitude coupling as oscillatory burst co-occurrence based on hidden Markov modeling (HMM) of MEG data. This unique combination of mathematical and neurobiological modeling allowed us to examine quantitatively how the power bias affects the connectivity spectrum of intrinsic functional networks and thereby demonstrate whether or not they reflect interacting neural oscillations.

## 2 Methods

### 2.1 MEG data acquisition

We analyzed 31 healthy right-handed adult volunteers (16 females; mean age: 26.4 years, range: 19–36 years; no history of neurological or psychiatric disease) taken from a MEG resting-state dataset used in previous publications (Mary et al., 2015;Vander Ghinst et al., 2016), including functional network mapping (Wens, Bourguignon, et al., 2014;Wens et al., 2019). Subjects signed a written informed consent and data usage was conformed to the HUB–Hôpital Erasme Ethics Committee approval (References: P2011/054, P2012/049). Neuromagnetic activity was recorded (analog band-pass: 0.1–330 Hz, sampling frequency: 1 kHz) during 5 min at rest, while subjects gazed at a fixation point, using a 306-channel whole-scalp-covering MEG system (Vectorview Neuromag, MEGIN) inside a lightweight magnetically shielded room (Maxshield, MEGIN; seeDe Tiège et al., 2008for details). Head movements were tracked with four head position indicator coils whose location relative to fiducials was digitized beforehand along with the face and scalp (Fastrack Polhemus). A standard brain 3D T1-weighted magnetic resonance image (MRI) was also acquired using a 1.5 T MRI scanner (Intera Philips) and co-registered manually with the head digitalization for individual head modeling and source reconstruction.

### 2.2 Data processing

Environmental interferences and head movements were suppressed using the temporal extension of signal space separation (Maxfilter v2.2 with default parameters, MEGIN;Taulu et al., 2005) and physiological interferences (cardiac and ocular), with an independent component analysis (FastICA of MEG signals filtered between 0.5 and 45 Hz and projected on their 30 principal components;Vigario et al., 2000). These data were then decomposed spectrally into narrow frequency bands (band-pass filter centers: 1, 2,…, 45 Hz, bandwidth: 1 Hz) and source projected by minimum norm estimation on a 5-mm grid covering the MRI brain volume (seeWens et al., 2015for implementational details).

Functional connectivity was estimated as the spectrally resolved AEC between pairs of source-projected MEG signals (Wens, Mary, et al., 2014; see alsoSI Theory), with geometric correction of spurious functional connectivity due to spatial leakage effects (Della Penna et al., 2019;Wens, 2015;Wens et al., 2015). Nodes of the interhemispheric connections between homologous cortices of primary networks (sensorimotor, SMN, MNI coordinates: left node, [–42,–26,54] mm, right node, [38,–32,48] mm; auditory, AN, left, [–54,–22,10] mm, right, [52,–24,12] mm; visual, VN, left, [–20,–86,10] mm, right, [16,–80,26] mm) were obtained from previous references (de Pasquale et al., 2012;Hipp et al., 2012). The functional connectome was estimated as the AEC between the 42 nodes of a point-wise resting-state network brain parcellation (de Pasquale et al., 2012). Since the geometric correction introduces small numerical asymmetries, any leakage-corrected AEC estimate between two source signalsxandywas symmetrized by averaging it with the corresponding AEC betweenyandx.

### 2.3 Power bias correction by renormalization

At the heart of our power bias correction is a mathematical model of amplitude coupling. We describe it fully inSI Theory. Here we summarize only the end result implemented in our MEG analysis pipeline. See alsoSI Methodsfor an alternative (though ultimately inadequate) correction based on standard regression modeling.

Starting from an estimate ofAECbetween the narrow-band MEG signalsxand leakage-corrected signaly, the true neural amplitude correlation (i.e., corrected for the power bias as well as for spurious contributions from noise amplitude correlations and linear synchronization, see below andSI Theory) may be recovered as the “renormalized” estimate

$${\text{AEC}}_{\text{ren}}=\frac{\text{AEC}-\frac{\text{cov}\left(A_{\in_{x}}^{2},A_{\in_{y}}^{2}\right)}{\sigma_{A_{x}^{2}}\sigma_{A_{y}^{2}}}-\frac{ℒ\left(x,y\right)+ℒ\left(x,\text{H}y\right)}{\sigma_{A_{x}^{2}}\sigma_{A_{y}^{2}}}}{\sqrt{1-{\left({\text{SNR}}_{x}^{\text{ampl}}\right)}^{-2}}\;\sqrt{1-{\left({\text{SNR}}_{y}^{\text{ampl}}\right)}^{-2}}}.$$

Like the original AEC, this quantity was eventually symmetrized overxandy. Here,$\in_{x}$and$\in_{y}$model “background noise processes”, i.e., the parts of signalsxandythat do not contribute to amplitude coupling (see below), andH ·andA·denote the complex Hilbert transform and its amplitude (Hilbert envelope). The denominator corresponds to the renormalization correcting the power bias itself; it depends on an amplitude-specific version

$${\text{SNR}}_{x}^{\text{ampl}}=\frac{\sigma_{A_{x}^{2}}}{\sqrt{\sigma_{A_{\in_{x}}^{2}}^{2}+2\langle A_{x}^{2}\rangle \langle A_{\in_{x}}^{2}\rangle -2{\langle A_{\in_{x}}^{2}\rangle }^{2}}}$$

of the signal-to-noise ratio (SNR, defined as${\text{SNR}}_{x}\;=$$\sigma_{x}\text{​}/\text{​}\sigma_{\in_{x}}$) and similar expressions for signaly(seeSI Theoryfor an explicit interpretation of${\text{SNR}}^{\text{ampl}}$). The second term in the numerator further corrects for noise amplitude correlations (although they were*a priori*expected to be subdominant by our definition of background noise, seeSI Theoryfor details andSI Resultsfor data-based evidence), making this power bias correction procedure valid even in the presence of correlated noise. The third and last term also corrects the effect of linear synchronization (*a priori*expected to be subdominant too due to leakage correction, seeSI TheoryandSI Results) embodied by the quantity

$$ℒ\left(x,y\right)=8\left[\text{cov}\left(x,y\right)\text{cov}\left(\in_{x},\in_{y\text{​}}\right)-\text{cov}{\left(\in_{x},\in_{y\text{​}}\right)}^{2}\right].$$

All temporal averages〈·〉—including standard deviationsσand covariancescov(·,·)—were estimated using all time points available in each individual recording. E.g., to model background noise as measurement noise, all statistics involving$\in_{x}$and$\in_{y}$were estimated from empty-room MEG signals at the brain locations corresponding to signalsxandy(5 min; processed with same filters, source projection, and leakage correction than subjects’ recordings).

### 2.4 Cerebral background noise model

Application of the above renormalization procedure requires to determine cerebral background noises$\in_{x}$and$\in_{y}$, i.e., neural processes not involved in amplitude coupling (seeSI Theoryfor a formal definition of background noise in the context of our mathematical model of AEC). It was modeled here as non-bursting brain activity (seeResultsfor further rationale) using a gaussian two-state HMM (HMM-MAR toolbox;Vidaurre, Abeysuriya, et al., 2018;Vidaurre et al., 2016) applied to the amplitude signal$A_{x}$(and leakage-corrected versions) at each brain node separately. Signal periods corresponding to non-bursting activity were inferred from the state showing the lowest mean amplitude with Viterbi decoding (Rabiner, 1989;Rezek & Roberts, 2005). Non-bursting power, non-bursting AEC, and all other statistics of$\in_{x}$and$\in_{y}$needed for power bias correction were computed by restricting time averages to coincident non-bursting periods both at signalsxand leakage-corrected signalsy. Non-bursting AEC was once again symmetrized overxandy. This enabled estimation of all the necessary statistical features of cerebral background noise, even though the HMM did not enable complete reconstruction of its time course simultaneously to the experimental recordings. We used non-bursting AEC estimates to provide explicit proof-of-concept that non-bursting activity does indeed properly model cerebral background noise in resting-state MEG data.

### 2.5 Synthetic electrophysiological signals

We simulated neural connectivity processes, i.e., the part of neural activity producing the amplitude coupling, as signal pairs$x_{0}$,$y_{0}$initially generated as independent band-filtered gaussian white noises (8–12 Hz; 1 kHz sampling rate; 5 min) and then mixed nonlinearly together in order to introduce a “neural” amplitude correlation${\text{AEC}}_{0}$. Specifically,$A_{y_{0}}$was replaced by$A_{y_{0}}\text{​}+kA_{x_{0}}$with

$$\begin{array}{c} k=\frac{{\text{AEC}}_{0}}{\sqrt{1-{\text{AEC}}_{0}^{2}}}\frac{\sigma_{A_{y_{0}}}}{\sigma_{A_{x_{0}}}}. \end{array}$$

Background noise processes$\in_{x}$,$\in_{y}$were simulated similarly with a background noise amplitude correlation${\text{AEC}}_{\text{noise}}$. Synthetic MEG signals were finally constructed by summing connectivity and noise processes, after independently rescaling them so as to fix SNR parameters. Simulations were run while controlling${\text{AEC}}_{0}$,${\text{AEC}}_{\text{noise}},$and SNR parameters, using time length and number of repetitions matched to the size of our experimental resting-state dataset. We restricted all simulations in the main text to the case${\text{AEC}}_{\text{noise}}=0$given the small levels of non-bursting AEC inferred from resting-state MEG data (which estimates${\text{AEC}}_{\text{noise}}$according to our cerebral background noise model described above). SeeSI Resultsfor example simulations involving non-zero noise coupling${\text{AEC}}_{\text{noise}}$.

### 2.6 Statistical procedures

The effect size of the power bias on amplitude correlation (power bias measure; PBM) was assessed as the relative difference between the estimatedAECand either the simulated coupling${\text{AEC}}_{0}$or the corrected estimate${\text{AEC}}_{\text{ren}}$in experimental MEG data, measured globally over the whole frequency range (1–45 Hz). Spectral similarities in either oscillatory power or amplitude connectivity were assessed statistically using one-sided Pearson correlation tests. The null distribution of Fisher-transformed correlationsRwas gaussian with mean 0 and variance1/(n−3), the numbernof spectral degrees of freedom being estimated from the cross-frequency covariance matrix of individual power or connectivity spectra (after averaging of the two spectra to be correlated). Significance was set top<0.05with the family-wise error rate controlled by Bonferroni correction for the number of independent nodesρestimated as the rank of the MEG forward model restricted to the nodes of the connectome (Wens et al., 2015) for power spectra, and for the numberρ(ρ−1)/​2of independent connections in the connectome (Sjøgård et al., 2019) for connectivity spectra. SeeSI Methodsfor full details.

## 3 Results

### 3.1 The power bias reflects SNR-dependent connectivity underestimation

**Fig. 1.**
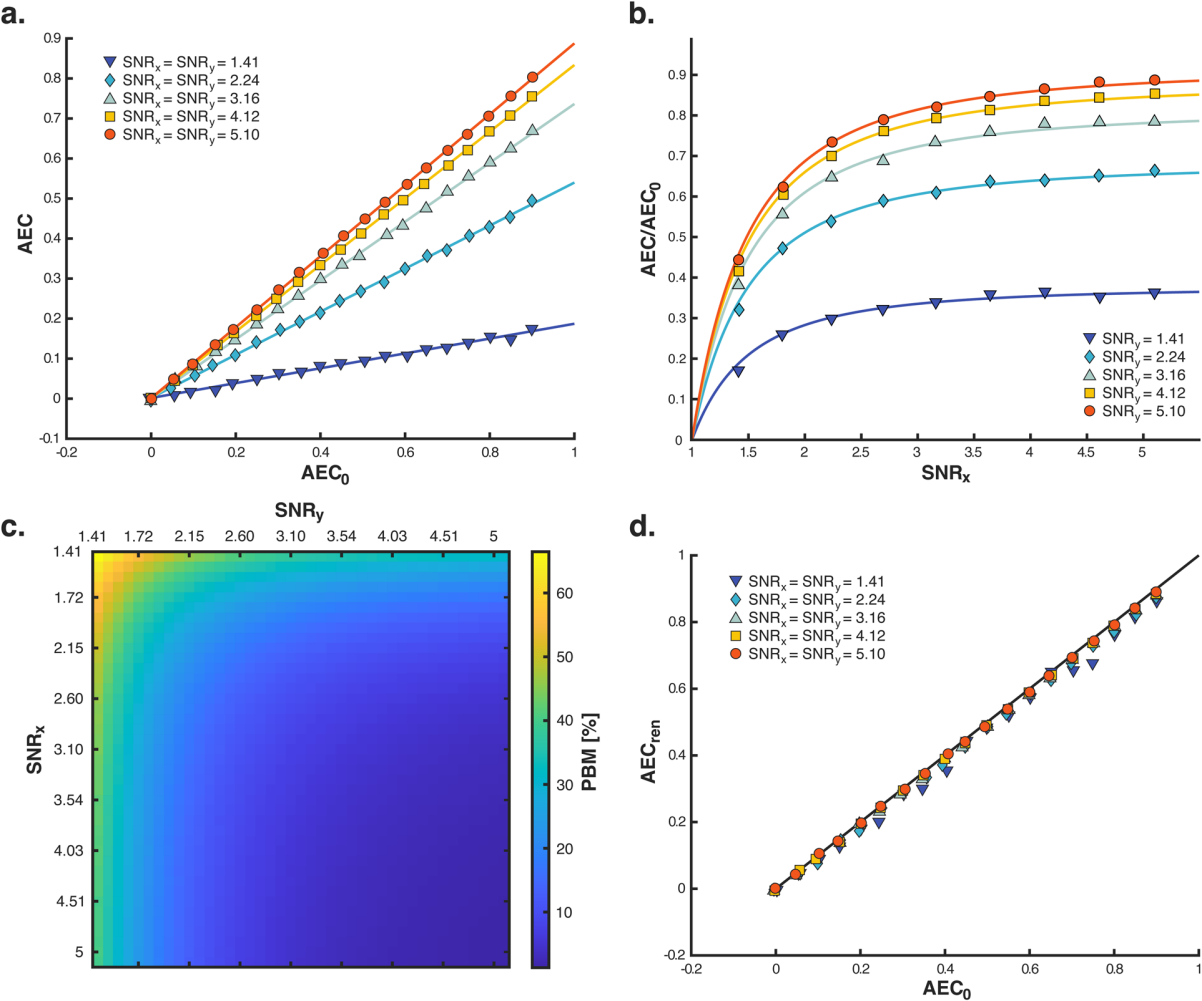
Power bias in amplitude correlation. Pairs of synthetic brain signals (x,y) were simulated at various SNR levels with independent noises and neural amplitude couplings (${\text{AEC}}_{0}$). (a) Linear relationship between AEC estimation and${\text{AEC}}_{0}$. Linear regression curves are superimposed to data points. (b) Nonlinear SNR dependence of the power bias, illustrated by plotting the slope ($\text{AEC}/{\text{AEC}}_{0}$) against the SNR. The superimposed model curves correspond to the nonlinearity${\left(1-2{\text{ SNR}}^{\text{-2}}\right)}^{1/2}$(seeSI Theory). (c) Deviation of AEC estimates from the neural amplitude coupling measured as a percentage (power bias measure, PBM; seeSI Methods) while systematically varying signal SNRs. (d) Connectivity corrected by renormalization (${\text{AEC}}_{\text{ren}}$) as a function of${\text{AEC}}_{0}$. The black diagonal line indicates perfect correction (${\text{AEC}}_{\text{ren}}={\text{AEC}}_{0}$). SeeSI Resultsfor an extension of this proof-of-concept to the case of correlated noise.

We started our study with a theoretical analysis of the power bias in AEC.Figure 1a–cillustrates the most salient features of the resulting theory. (SeeMethodsfor a detailed formulation of the theory, andSI Theoryfor mathematical derivations.) We simulated pairs of synthetic electrophysiological signals mixing “connectivity processes,” i.e., the part of neural activity that generates the amplitude coupling, and “cerebral background noise processes,” i.e., the part of neural activity that does not participate to this coupling (seeSI Theoryfor a formal definition). Simulations were performed at various levels of neural amplitude coupling and of SNR, which turned out to be the main parameter controlling the power bias (seeMethods). The relationship between AEC estimated from electrophysiological signals and the underlying neural amplitude coupling (${\text{AEC}}_{0}$) was linear with a slope that decreased when lowering the SNR (Fig. 1a). The slope was close to 1 at high SNR, indicating accurate amplitude coupling estimation when connectivity processes dominate over background noise ($\text{AEC}\approx {\text{AEC}}_{0}$); but then it decreased as the SNR got lower, revealing connectivity underestimation due to background noise. This SNR-dependent underestimation corresponds precisely to the power bias (Hipp & Siegel, 2015;Muthukumaraswamy & Singh, 2011).

### 3.2 A key characteristic of the power bias is its nonlinearity in the SNR

Figure 1bexamines the slope mentioned above against the SNR of one signal. Connectivity raised sharply from 0 at small SNR, where neural amplitude coupling is undetected because noise dominates over connectivity processes, and plateaued at large SNR where functional connectivity is detected—though still possibly underestimated due to noise in the other signal. The SNR of the second signal modulated this plateau in a similar nonlinear fashion. Unbiased estimation of neural connectivity was only reached when both SNRs were large enough. The combined effect of both SNR nonlinearities enables to assess the effect size of the AEC power bias quantitatively (Fig. 1c). EstimatedAECdeviated from the simulated neural coupling${\text{AEC}}_{0}$by 60% when the two signals exhibited a low SNR of 1.4, but this deviation decreased rapidly at higher SNRs to reach below 10% when both SNRs were above 3. We conclude that the AEC power bias becomes negligible once the SNR exceeds 3.

Of note, our general analysis of AEC disclosed two other sources of bias, i.e., background noise amplitude correlations and zero-lag synchronization processes; however, both of them were*a priori*expected to be subdominant compared with the power bias itself. SeeMethodsandSI Theoryfor details, andSI Resultsfor data-based evidence.

### 3.3 The power bias may be corrected by renormalizing AEC estimates

Figure 1asuggests that the underestimation effect of the power bias may be corrected by renormalizingAECwith the nonlinear slope factor illustrated inFigure 1b(seeMethods).Figure 1dprovides proof-of-concept for this procedure using synthetic signals with time length and frequency content commensurate to the experimental MEG recordings analyzed below. The renormalization allowed to successfully recover the simulated neural amplitude coupling with high accuracy, even at the lowest SNRs (correction error=0.4%atSNR=1.4;<0.1%forSNR>3).

Of note, the simulations inFigure 1were restricted to the case of independent noises given the negligible amount of background noise correlations in the MEG resting-state data analyzed below. Nevertheless, the full renormalization procedure also allows to control for possible noise dependencies (seeMethods). SeeSI Resultsfor an extension of the proof-of-conceptFigure 1din the presence of correlated noises.

### 3.4 Cerebral background noise may be identified as a non-bursting brain state

**Fig. 2.**
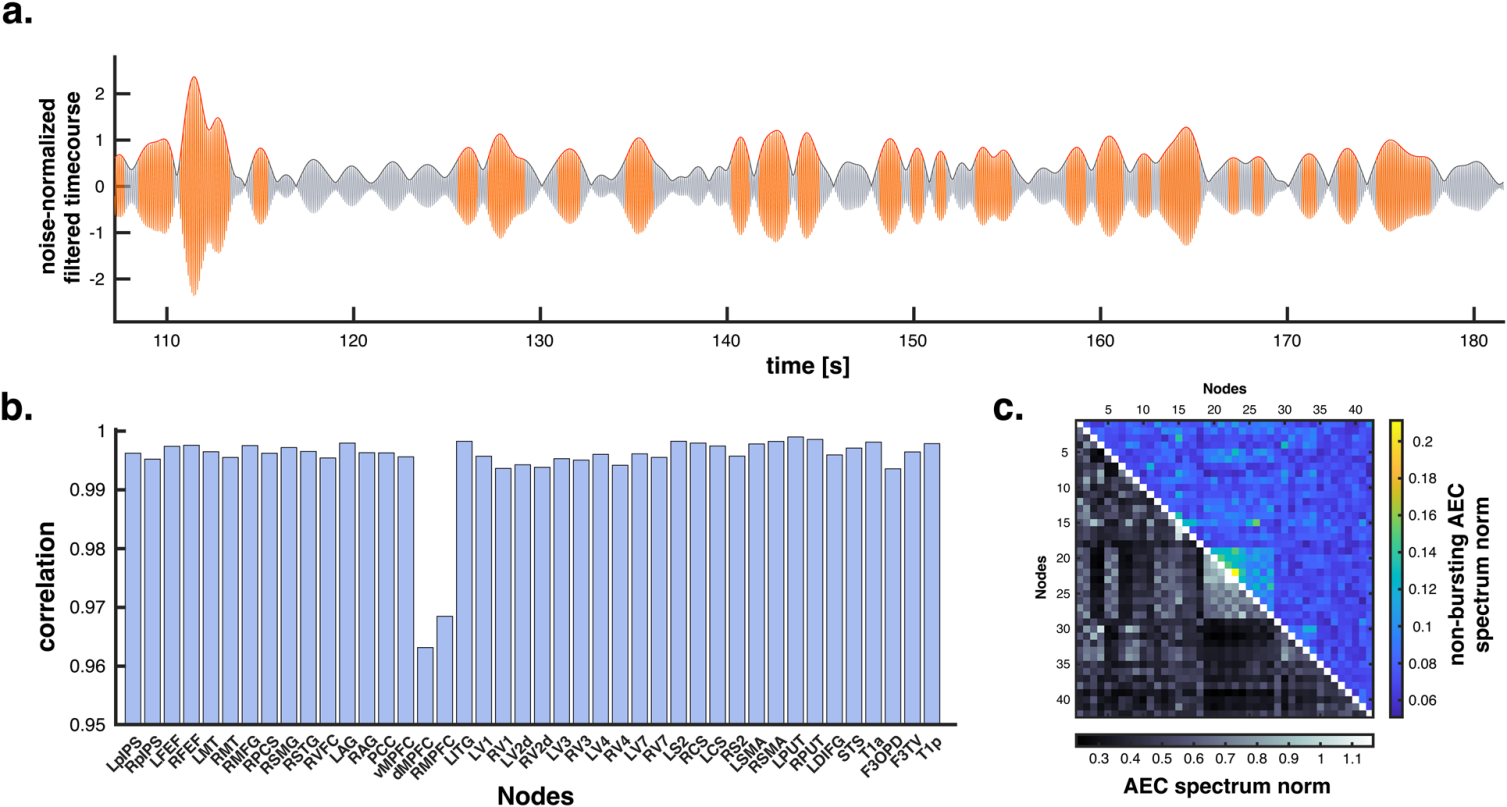
Cerebral background noise model based on non-bursting activity. (a) Illustration of the bursting (orange) and non-bursting (gray) states determined by the HMM applied to the MEG signal in the left sensorimotor network (node MNI coordinates, [-42, -26, 54] mm) filtered in the narrow [9.5, 10.5] Hz band. (b) Spectral similarity of power spectra computed on the entire signal and non-bursting power spectra at the corresponding node, assessed using Pearson correlation. For explicit illustrations, seeFigure 3, bottom, blue and green spectra. (c) Norm of non-bursting AEC spectra (upper right triangle) compared with AEC spectra computed on the entire signals (lower left triangle). The smallness of non-bursting AEC norms indicates that non-bursting activity does not exhibit amplitude coupling and thus properly models cerebral background noise. For explicit illustrations, seeFigure 3, top, black spectra.

To apply our renormalization procedure to experimental MEG functional connectivity data, we first had to determine what the theoretical notion of “cerebral background noise” in our model (seeSI Theoryfor general conditions defining consistent noise models) represents in our electrophysiological recordings. The problem was, therefore, to identify and isolate resting-state MEG signals exhibiting no AEC. Given the neurobiological finding that AEC mostly reflects the coincident bursting of brain oscillations (Seedat et al., 2020), we tried and modeled cerebral background noise explicitly as non-bursting brain activity (Fig. 2). We identified non-bursting periods of MEG recordings as the low-amplitude state of a HMM applied locally to each brain node of the connectome (seeFig. 2afor an illustration). Comparison of oscillatory power spectra estimated using the entire signal at each node and of power spectra restricted to the corresponding non-bursting state activity revealed strong spectral similarities across the whole brain (regularized Pearson correlation test; allRs>0.96,$p\text{s}<10^{-4}$controlling for the family-wise error rate;Fig. 2b). This demonstrates that non-bursting activity is oscillatory. Non-bursting AEC, i.e., AEC restricted to coincident non-bursting state activity in two brain nodes, revealed flat connectivity spectra and low connectivity levels across the whole connectome (Fig. 2c). This means that non-bursting oscillations do not contribute to amplitude coupling (confirming that it is driven by bursting activity), and, therefore, that they provide an adequate model of cerebral background noise to investigate the power bias in AEC.

**Fig. 3.**
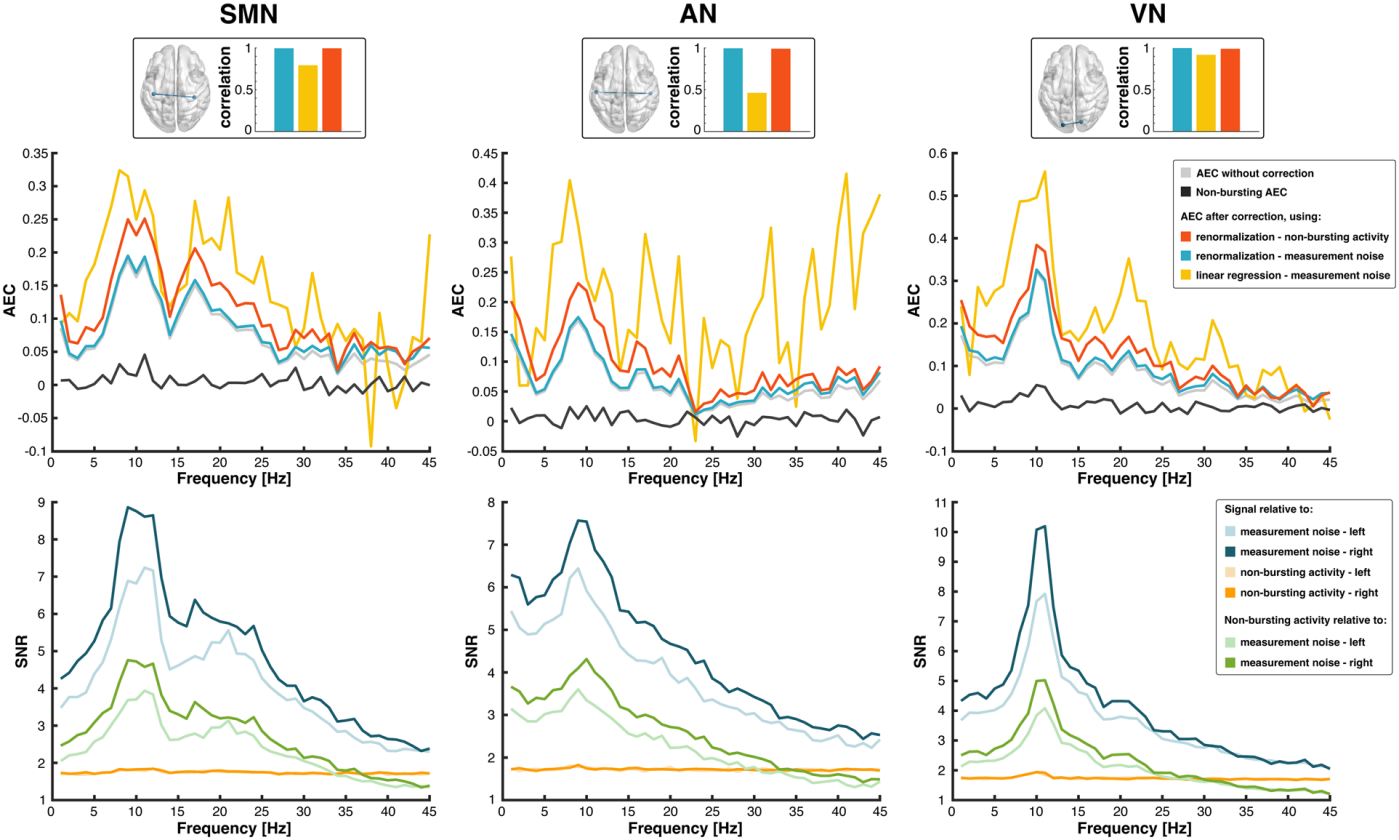
Power bias correction in interhemispheric amplitude correlation spectra. Top: Spectrally resolved AEC is shown for three interhemispheric functional connections without correction (gray), after power bias correction based on AEC renormalization relative to cerebral background noise modeled as a non-bursting brain state (red) or to measurement noise (blue), and after linear regression (yellow). The AEC spectra of corresponding non-bursting activity are also shown (black; see alsoFig. 2cfor a summary measure of the spectral flatness of non-bursting AEC). Inserts show the connections on the MNI glass brain and bar charts comparing levels of AEC spectral similarities (Pearson correlation between non-corrected and corrected AEC spectra). Bottom: Spectrally resolved SNR of MEG signals at the two nodes of each connection relative to cerebral background noise (orange) or to measurement noise (blue), and of non-bursting activity relative to measurement noise (green).

To illustrate these observations, we analyzed interhemispheric AEC in three low-level brain networks (SMN, AN, VN)—arguably the clearest hallmark of electrophysiological brain networks (Fig. 3, top). Their connectivity spectra exhibited typical peaks in theαand theβfrequency bands (gray spectra), but they were flat for non-bursting AEC (black spectra), in line withFigure 2c. This was in stark contrast with the non-bursting power spectra at the corresponding nodes (Fig. 3, bottom, green), which showed the same peaks than the oscillatory power spectra (blue), in line withFigure 2b.

### 3.5 The spectral content of interhemispheric connectivity networks is preserved by power bias correction

Figure 3illustrates the fact that AEC spectral peaks (Fig. 3, top, gray) coincide with the presence of rhythmic brain activity at the corresponding nodes (Fig. 3, bottom, blue), which warrants a proper analysis of the power bias. The SNR relative to cerebral background noise (orange spectra) ranged from 1.7 to 2, for which simulations predicted AEC underestimation with effect size betweenPBM≈5%and30%(Fig. 1cat corresponding synthetic “gaussian” SNRs ranging from 2 to 3.5; seeSI Theoryfor details on the link between experimental SNR and synthetic “gaussian” SNR). Accordingly, power bias correction leveled up AEC (Fig. 3, top, red spectra) with effect sizes in the expected range (PBM values: SMN, 18%; AN, 26%; VN, 17%). Crucially, the SNR relative to cerebral background noise still exhibited discernableα- andβ-rhythm peaks reflecting oscillatory bursts, but the shape and peaks of AEC spectra were preserved after correction (regularized Pearson correlation test; SMN,R=0.996; AN,R=0.991; VN,R=0.990). This confirms the physiological role ofα- andβ-bursts in the emergence of primary functional networks as their AEC cannot be solely accounted for by their high spectral power.

We further assessed to what extent this conclusion depends on our model of cerebral background noise by reanalyzing power bias correction, only this time relative to measurement noise. This would amount to assume that the entirety of cerebral activity participates to amplitude coupling. In this case too, AEC spectra were preserved after correction (Fig. 3, top, blue;PBM=1%andRs>0.995for all three connections), consistently with the mixture of high (SNR=2/“gaussian”SNR=3, corresponding to predictedPBMbelow 10%) to very high SNR values relative to measurement noise (SNR=10/“gaussian”SNR=16in theαband, corresponding to predictedPBMwell below 1%;Fig. 3, bottom, blue spectra). On the other hand, the SNR nonlinearity of our model turned out to be key to draw our conclusion. Indeed, standard linear regression modeling proved inadequate for power bias correction (Fig. 3, top, yellow; seeSI Resultsfor more details).

### 3.6 These conclusions generalize to the whole electrophysiological connectome

**Fig. 4.**
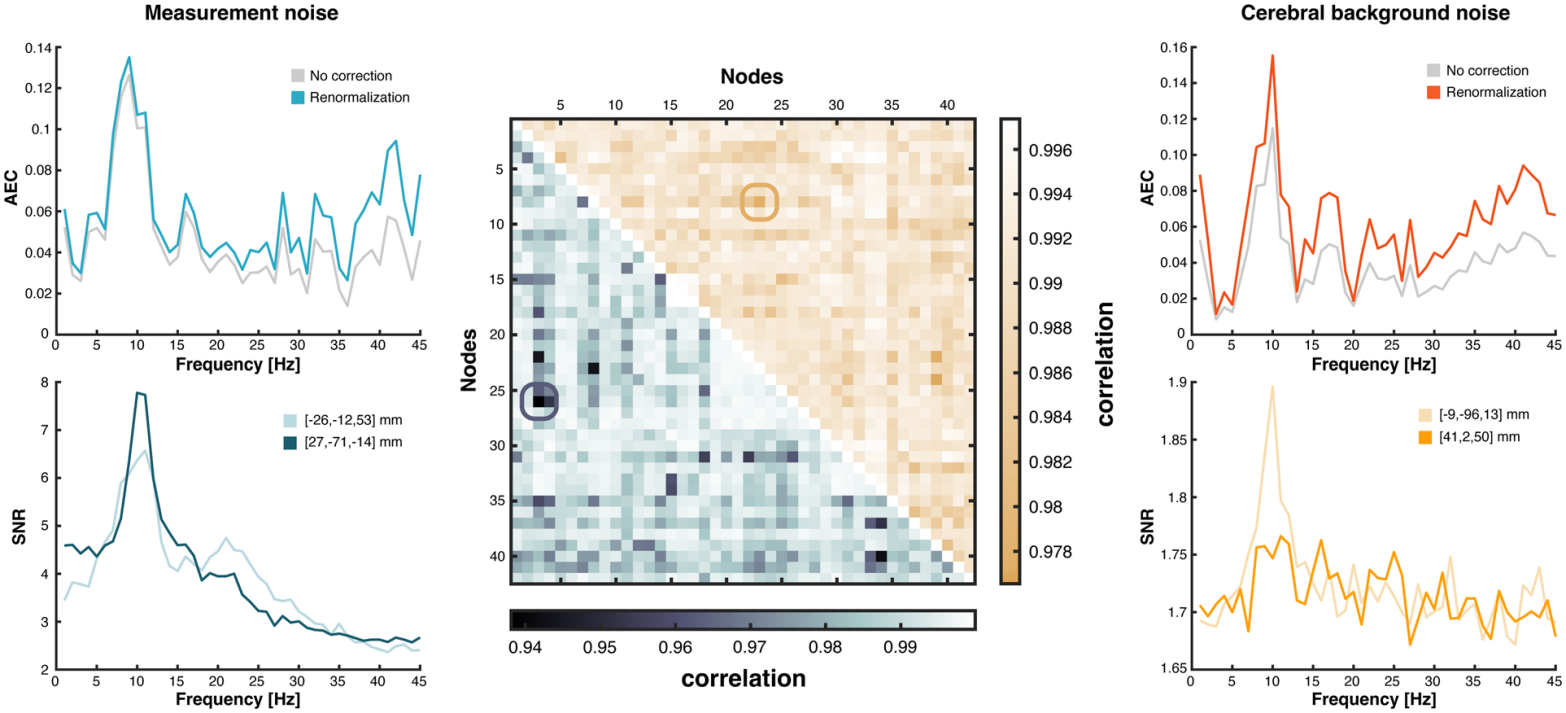
Power bias in amplitude correlation spectra of the connectome. Middle: The spectral deformation effect on AEC spectra due to power bias correction relative to cerebral background noise (upper right triangle) or to measurement noise (lower left triangle) was mapped in a 42-node whole-brain-covering connectome (de Pasquale et al., 2012) using a matrix of Pearson correlations between the non-corrected and corrected AEC spectra. Left: Functional connectivity (top) and SNR (bottom) spectra of the connection with the worst spectral deformation when modeling noise as measurement noise. Its location in the connectome is highlighted in the corresponding correlation matrix. Right: Same as left, but modeling cerebral background noise as non-bursting brain activity.

We then investigated AEC spectral deformations associated with the power bias systematically across the whole brain connectome (Fig. 4). Whatever the type of noise model considered in the correction, the shape of connectivity spectra was not deformed (regularized Pearson correlation, allRs>0.938,$p\text{s}<210^{-13}$after controlling for the connectome-level family-wise error rate). The least similar spectra were located at a connection linking the visual (node MNI coordinates, [-9, -96, 13] mm) and the ventral attention networks ([41, 2, 50] mm) for power bias correction relative to cerebral background noise (Fig. 4, right;R=0.977), and at a connection linking the dorsal attention ([-26, -12, 53] mm) and the visual networks ([27, -71, -14] mm) for power bias correction relative to measurement noise (Fig. 4, left;R=0.938). Inspection of these worst-case examples shows that spectral peaks in AEC connectivity are indeed all preserved after correction. We conclude that the power bias does not impact spectral features of intrinsic functional connectivity.

## 4 Discussion

Combining a rigorous mathematical model of the power bias in frequency-dependent amplitude coupling with a biologically informed model of cerebral background noise as non-bursting brain oscillations, we demonstrated that the spectral content of resting-state MEG amplitude connectivity is preserved after power bias correction, despite fairly low SNRs relative to cerebral background noise. This provides strong empirical evidence that intrinsic functional network connectivity does reflect, from an electrophysiological standpoint, couplings among spontaneous brain rhythms.

### 4.1 Intrinsic functional networks reflect interacting brain rhythms

The oscillatory nature of intrinsic functional connectivity at rest has been taken for granted since the first successful electrophysiological mappings of spectrally resolved brain networks with MEG (Brookes et al., 2011;Hipp et al., 2012;Liu et al., 2010), and was actually considered well before (Leopold et al., 2003). Still, the effect of the power bias (Muthukumaraswamy & Singh, 2011) was not controlled explicitly, leaving the door open that functional connectivity may not be specific to rhythmic activity (Hipp & Siegel, 2015). A direct consequence of our analysis is that intrinsic functional networks do correspond to interacting neural oscillations.

Our experimental evidence provides independent confirmation of the consensus on what are the neurophysiological mechanisms of intrinsic functional connectivity revealed by resting-state amplitude correlations (Cabral, Kringelbach, et al., 2014;Deco et al., 2011). Large-scale neurocomputational models seeking to describe the underlying biophysics often share the same spirit (notwithstanding differences in their details). They start with localγ-band oscillations generated within each isolated cortical column, e.g., through feedback loops between excitatory and inhibitory neurons (Wilson & Cowan, 1972). These fast, local oscillations then interact via long-range excitatory synaptic couplings supported by the anatomical circuitry that connects remote populations. This results into a collective, brain-wide dynamics from which network-level brain rhythms unfold at lower frequencies, mostly in the slowerθ,α,andβbands due to conduction delays (Cabral et al., 2017;Cabral, Luckhoo, et al., 2014;Tewarie et al., 2019). In this framework, functional connectivity is thus an emergent dynamical phenomenon that can be described in terms of interacting neural oscillations. The significance of our data is to set this theoretical framework on firmer grounds. Large-scale neurocomputational models scarcely generate these local cortical oscillations from detailed neural membrane dynamics but rather use them as a starting point, either by modeling neural populations explicitly as oscillators with Kuramoto-like models (Breakspear et al., 2010;Cabral et al., 2011;Cabral, Luckhoo, et al., 2014), or as neural mass models with parameters chosen to exhibit a limit cycle or at least to stand at the brink of its bifurcation (Deco et al., 2009;Tewarie et al., 2019;Wilson & Cowan, 1972). Admittedly, population-level oscillations are generically observed in local, fully connected networks of spiking neurons (Gerstner et al., 1996); still other possibilities involving irregular, chaotic behavior have been described in local networks exhibiting excitation–inhibition balance (Brunel, 2000;Brunel & Wang, 2003;van Vreeswijk & Sompolinsky, 1996). Had power bias correction demonstrated a broadband functional connectivity, the latter possibility could have been reconsidered. In fact, chaotic or stochastically driven models have been used in the context of fMRI connectivity (Deco et al., 2009;Honey et al., 2007), but to our knowledge, their consequence at the level of MEG functional connectivity has not been investigated. Our result provides independent empirical data that such revision is, fortunately, not warranted. The conceptual framework based on the interplay between locally generated neural oscillations and large-scale delayed interactions thus stands.

It is fair to mention at this point a methodological limitation of our MEG analysis framework, and of functional connectivity in general for that matter. Connectivity estimation provides only an indirect way to probe the neural interactions occurring in the brain and may be fraught with several interpretation issues, of which the power bias is but one example (S. Palva & Palva, 2012). In particular, functional connectivity measures may contain “ghost interactions” related to secondary spatial leakage effects and uncertainties in the exact location of nodes in the functional connectome (Colclough et al., 2015;J. M. Palva et al., 2018;Wens, 2015;Wens et al., 2015). This being said, most issues relate to spatial deformations rather than spectral deformations*per se*; e.g., they would lead to an artefactual spread of oscillatory connectivity across the connectome through ghost interactions. The persistence of the spectral content of amplitude connectivity after our power bias correction thus still provides evidence for the existence of a close relationship between interacting neural oscillations and intrinsic functional networks.

### 4.2 Spontaneous brain activity contains non-bursting “background” oscillations unrelated to amplitude coupling

The next question is to know what kind of neural oscillation supports the functional binding of neural assemblies into intrinsic networks. Previous work established a key connection between amplitude connectivity at rest and transient oscillatory bursts (Seedat et al., 2020). These bursts emerge naturally in neurocomputational models near criticality, i.e., close to bifurcations of limit cycles, and indicate the presence of metastable brain oscillations (Hindriks & Tewarie, 2023;Freyer et al., 2011). On the other hand, the debate remains open on whether oscillatory bursts really differ from sustained brain rhythms, both from the electrophysiological and the functional perspectives (van Ede et al., 2018). Our biologically informed model of background noise in amplitude coupling brings further insight into this question.

While our study was not initially focused on the role of oscillatory bursts in functional brain networks, in the course of our analysis, we used non-bursting activity to identify the part of neural signals disengaged from amplitude connectivity. We split our resting-state MEG data into a bursting phase characterized by transient increases of oscillatory amplitude, and a non-bursting phase characterized by lower levels of oscillatory amplitude. The non-bursting phase itself turned out to be oscillatory and to contain dominant, sustained rhythms in theαand theβfrequency bands, but crucially it did not exhibit any amplitude correlation. This suggests that spontaneous brain activity contains two functionally distinct classes of spectrally similar neural oscillations: transient oscillatory bursts subtending amplitude connectivity (Seedat et al., 2020), and non-bursting background oscillations not involved in the process of amplitude coupling. The idea of considering brain rhythms as being composed of transient bursts has gained significant weight over the last years (Jones, 2016;van Ede et al., 2018) and its functional implications received a lot of attention, be it in relation to motor control (Bonaiuto et al., 2021;Feingold et al., 2015;Sherman et al., 2016), working memory (Higgins et al., 2021;Lundqvist et al., 2016), or resting-state functional connectivity (Baker et al., 2014;Coquelet et al., 2022;Hindriks & Tewarie, 2023;Seedat et al., 2020;Vidaurre, Hunt, et al., 2018). On the other hand, the possibility and functional implications of non-bursting, possibly sustained, brain oscillations remain largely unexplored. Our usage of this concept was restricted here to the analysis of the power bias in amplitude connectivity, in which non-bursting oscillations are reduced to a mere “cerebral background noise”. Still, this terminology should not mislead us to think that they genuinely lack any functional relevance (quite analogously to how, in the past, spontaneous brain activity was discarded as unstructured brain noise in the pre-resting-state era; see, e.g.,Deco & Corbetta, 2011). In fact, we envision that a larger field of research might develop around the study of non-bursting brain oscillations.

### 4.3 Bursting oscillations provide a physiological link between oscillatory power and amplitude connectivity

Even though non-bursting background oscillations do not participate to amplitude coupling*per se*, they do affect amplitude connectivity estimation by playing the role of “cerebral background noise” in the power bias, i.e., noise with respect to the neural processes generating amplitude coupling. In fact, this allows to give a direct physiological interpretation to the power bias in amplitude correlation. It stands to reason that a brain activity exhibiting oscillatory bursts that are too rare or not ample enough (i.e., low SNR in our model) cannot be well discriminated from non-bursting oscillations and will thus show poor sensitivity to burst-dependent amplitude coupling, i.e., connectivity underestimation. Non-bursting oscillations could thus theoretically lead to connectivity spectral deformations, mostly outside theαandβfrequency bands where oscillatory bursts are best identified (Seedat et al., 2020), and to a flattening of the connectivity spectrum after power bias correction. Our model attempted to properly disentangle the impact of non-bursting oscillations from the actual amplitude coupling generated by bursting oscillations. It did not reveal spectral deformations but rather demonstrated the presence ofαandβspectral peaks in amplitude connectivity. This provides evidence that amplitude coupling is predominantly carried byαandβbursts rather than oscillatory bursts in other frequency bands (Seedat et al., 2020).

In this context, the spectral similarity between oscillatory power and functional connectivity appears to be physiological and not artefactual; both reflect the spectral content of their common denominator—the oscillatory bursts. That is why the broadband functional connectivity hypothesis ofHipp and Siegel (2015)may not be warranted after all. This hypothesis was based on the fairly reasonable assumption that spectral coincidences between measures of functional connectivity estimation and SNR must be the result of an artifact (i.e., the power bias). Our careful modeling of amplitude coupling suggests that they do reflect physiology, but it also highlighted how non-trivial it would be to reach this conclusion without the analysis tools developed here. Our model explains how spectral similarities between power (dominated by the contribution of non-bursting oscillations) and amplitude connectivity (driven by oscillatory bursts) largely reflect spectral similarities between non-bursting and bursting oscillations, but the latter coincidence remains unexplained at the moment. Following the abovementioned framework based on large-scale delayed interactions, a possible reason could be that non-bursting and bursting oscillations are generated by neural circuits with similar geometry and delays (leading to similar spectral content;Tewarie et al., 2019) but distinct stability properties (Freyer et al., 2011). However, pursuing this question goes beyond the reach of our analysis.

Another question that our analysis cannot elucidate is, what would be the possible function of this physiological relationship between oscillatory burst power and functional connectivity.Daffertshofer and van Wijk (2011)demonstrated explicitly how local oscillatory power may influence long-range synchronization through a purely neurodynamical mechanism. This was also illustrated byTewarie et al. (2018)using simulated biophysical models. Interestingly, the latter work included discussion of the power bias in MEG connectivity; the authors argued that the relationship observed between power and phase couplings in MEG data is physiological because models do exhibit such relationship. Providing a direct proof of this claim would require extending our model to the context of phase connectivity, which represents a challenging avenue for future work (seeSI Theory). This would be particularly valuable to try and dissociate the possibly distinct contributions of bursting and non-bursting oscillations to neural phase synchronization, and eventually to interpret rigorously findings of transient phase couplings based on time-embedded HMM of resting-state MEG recordings (which is partially confounded by power;Vidaurre, Hunt, et al., 2018).

### 4.4 Power bias correction by renormalization may become an important tool to interpret electrophysiological network connectivity at rest and in task

We investigated here specifically the oscillatory nature of intrinsic brain networks, but our power bias correction may also prove useful in cognitive neuroscience applications seeking to assess the impact of different tasks, behavioral conditions, or populations on functional connectivity (Muthukumaraswamy & Singh, 2011). Even though non-bursting oscillations do not affect the spectral features of amplitude connectivity, we observed underestimation effects that could reach substantial levels (up toPBM=33%change after power bias correction; though this effect did not reach statistical significance, seeSI Results). Our renormalization procedure allows precisely to infer what the burst-dependent amplitude correlation would be without the disturbances brought by non-bursting activity (which by the way makes our renormalized amplitude connectivity close in spirit to other burst-related connectivity measures such as burst coincidence,Seedat et al., 2020, or co-kurtosis,Hindriks & Tewarie, 2023). We conclude that power bias correction may be essential to properly interpret functional connectivity contrasts across behavioral or clinical conditions, especially in situations where brain rhythms are altered. Possibilities include, e.g., task-induced desynchronizations (Pfurtscheller & Lopes Da Silva, 1999), pharmacological treatments (Leong et al., 2022), and brain pathologies (Babiloni et al., 2021).

We should keep in mind though that the approach developed here may break down in situations where brain rhythms are suppressed too strongly. This is because cerebral noise modeling based on the HMM would fail to discriminate low- and high-amplitude signal periods in the absence of bursting oscillations, leading to low SNR estimates and ill-conditioning of the renormalization procedure (seeSI Theoryfor further details). That being said, it is actually the whole paradigm of burst-dependent functional connectivity that loses its meaning then, not just our power bias correction. This limitation of the renormalized amplitude correlation technique also prompts the question of whether alternatives exist for a systematic correction of the power bias. Standard techniques based on within-condition power regression of functional connectivity data proved inadequate due to the inherent nonlinearity of the power bias in the SNR (seeSI Results). This further emphasizes the crucial role of SNR nonlinearity in our model as well as the necessity of using renormalization instead of regression. In hindsight, this failure is the reason why in past publications, we could only rely on regression designs that suppress specifically the effect of between-condition power changes in corresponding connectivity differences without modeling its effect within each condition (Naeije et al., 2019;Sjøgård et al., 2020;Van Dyck et al., 2021a). Investigating the impact of power bias correction on functional connectivity contrasts across physiological or pathological conditions—and how this affects statistical inferences and interpretations—thus represents an important avenue for future work in the context of cognitive and clinical applications.

In conclusion, our analysis of brain amplitude couplings allowed not only to prove the neural oscillation theory of intrinsic functional network connectivity, but also to disentangle the role of bursting and non-bursting oscillations. Further, it demonstrates the usefulness of moving away from generic linear modeling and toward theoretical developments that integrate rigorous mathematical analysis to neurobiologically informed models of brain dynamics.

## Supplementary Material

Supplementary Material

## Data Availability

Data and code will be made available upon request to the corresponding author and with the approval of institutional authorities (CUB – Hôpital Erasme and Université libre de Bruxelles).
