# Supplementary material for: The dissociative role of bursting and non-bursting neural activity in the oscillatory nature of functional brain networks
Source: Imaging Neurosci (Camb). 2024 Jul 17;2:imag-2-00231. doi: 10.1162/imag_a_00231 (PMC12272262; doi:10.1162/imag_a_00231)
Supplement: Supplementary Material [file imag_a_00231-supp.pdf]

Supplemental Information

The dissociative role of bursting and non-bursting neural activity in the oscillatory nature of functional brain networks

Alix Cordier[a], Alison Mary[b], Marc Vander Ghinst[a,c], Serge Goldman[a,d], Xavier De Tiège[a,e], Vincent Wens[a,e]

[a] *Université libre de Bruxelles (ULB), ULB Neuroscience Institute (UNI), Laboratoire de Neuroanatomie et Neuroimagerie translationnelles (LN²T), Brussels, Belgium*
[b] *Université libre de Bruxelles (ULB), ULB Neuroscience Institute (UNI), Neuropsychology and Functional Neuroimaging Research Unit (UR2NF) at Centre de Recherches Cognition et Neurosciences (CRCN), Brussels, Belgium*
[c] *Université libre de Bruxelles (ULB), Hôpital Universitaire de Bruxelles (HUB), CUB Hôpital Erasme, Department of Ear, Nose and Throat, and of Cervico-facial surgery, Brussels, Belgium*
[d] *Université libre de Bruxelles (ULB), Hôpital Universitaire de Bruxelles (HUB), CUB Hôpital Erasme, Department of Nuclear Medicine, Brussels, Belgium*
[e] *Université libre de Bruxelles (ULB), Hôpital Universitaire de Bruxelles (HUB), CUB Hôpital Erasme, Department of Translational Neuroimaging, Brussels, Belgium*

Appendix A. SI Theory

We develop here in detail our mathematical theory of the power bias in spectrally resolved amplitude correlation. As a preliminary, we start with the simplest case of linear correlation, as it allows to illustrate clearly key theoretical features of the connectivity power bias, how a renormalization of functional connectivity enables to correct the bias, and what extra modeling steps are required for its implementation in practice.

Appendix A.1. Warm-up: Linear correlation

Setup. The linear correlation R between two signals $x = x(t)$ and $y = y(t)$ is given by

$$R = \mathrm{corr}[x, y] = \frac{\mathrm{cov}(x, y)}{\sigma_x\, \sigma_y} \cdot \tag{1}$$

This corresponds to the covariance $\mathrm{cov}(x, y) = \langle xy\rangle - \langle x\rangle\langle y\rangle$ of x and y suitably normalized by their standard deviation $\sigma_x = \sqrt{\langle x^2\rangle - \langle x\rangle^2}$ and $\sigma_y = \sqrt{\langle y^2\rangle - \langle y\rangle^2}$. Brackets $\langle\cdot\rangle$ denote time averaging. In our context, x and y represent experimental MEG signals reconstructed at two distinct brain locations. They can be decomposed as sums of the neural connectivity processes (respectively denoted hereafter as x_0 and y_0) that subtend the observed linear coupling, and of background noise (respectively ϵ_x and ϵ_y) that may be of neural origin but do not participate to the generation of this coupling. Explicitly,

$$x = x_0 + \epsilon_x\,, \qquad y = y_0 + \epsilon_y\,. \tag{2}$$

These simple-looking equations entail the hidden assumption that x_0 and y_0 are not mixed, as would be expected after source projection due to the spatial leakage effect (Wens, 2015). We shall keep this assumption since all our connectivity estimations included spatial leakage correction beforehand (Wens et al., 2015), notwithstanding possible remaining ghost interactions (Palva et al., 2018; see also main text for a discussion).

Our goal is to express the measured correlation (1) in terms of the neural coupling

$$R_0 = \mathrm{corr}\,[x_0, y_0] = \frac{\mathrm{cov}\,(x_0, y_0)}{\sigma_{x_0}\,\sigma_{y_0}} \tag{3}$$

and the SNR estimates

$$\mathrm{SNR}_x = \frac{\sigma_x}{\sigma_{\epsilon_x}}\,, \qquad \mathrm{SNR}_y = \frac{\sigma_y}{\sigma_{\epsilon_y}} \cdot \tag{4}$$

To keep the model as general as possible, we also leave the possibility of correlations $R_{\mathrm{noise}} = \mathrm{corr}[\epsilon_x, \epsilon_y]$ among noises.

Power bias in linear connectivity. We demonstrate below that the above setup leads to the relationship

$$R = R_0\, \sqrt{1 - \mathrm{SNR}_x^{-2}}\, \sqrt{1 - \mathrm{SNR}_y^{-2}} + \frac{R_{\mathrm{noise}}}{\mathrm{SNR}_x\,\mathrm{SNR}_y} \cdot \tag{5}$$

The correlation R can thus be decomposed into two distinct terms. The first encapsulates the power bias phenomenon and illustrates general features that we discuss now, momentarily neglecting noise correlations (i.e., we set $R_{\mathrm{noise}} = 0$ in Eq. 5). First, the power bias leads to an underestimation of functional connectivity magnitude, $|R| < |R_0|$. This effect is worst when noise dominates over connectivity processes ($\mathrm{SNR}_x \approx 1$ or $\mathrm{SNR}_y \approx 1$, so $R \approx 0$) but is negligible when connectivity processes dominate ($\mathrm{SNR}_x \gg 1$ and $\mathrm{SNR}_y \gg 1$, so $R \approx R_0$). Second, this underestimation occurs through a SNR-dependent multiplicative factor. This is precisely what allows to disentangle the power bias from genuine functional connectivity, which can be recovered as a suitably renormalized version of the connectivity measure.

*Corresponding author. *Address:* Department of Translational Neuroimaging, CUB Hôpital Erasme, 808 Route de Lennik, 1070 Brussels, Belgium. *E-mail address:* alix.cordier@ulb.be.

Power bias correction by renormalization. In the present case, and restoring the possibility of noise correlations, the *renormalized* correlation

$$R_{\text{ren}} = \frac{R - \frac{R_{\text{noise}}}{\text{SNR}_x\,\text{SNR}_y}}{\sqrt{1 - \text{SNR}_x^{-2}}\,\sqrt{1 - \text{SNR}_y^{-2}}} \tag{6}$$

indeed allows to recover the neural correlation, $R_{\text{ren}} = R_0$. Of notice, the renormalization factor (i.e., the denominator in Eq. 6) depends nonlinearly on the SNRs, indicating that conventional multiple regression modeling may not be able to efficiently correct the power bias. Numerically, the division entailed by the renormalization (6) must be well conditioned. This is not the case in the limit of low SNR ($\text{SNR}_x \approx 1$ or $\text{SNR}_y \approx 1$), for small estimation errors in the SNR estimates (4) then translate into large errors in the connectivity estimate (6). Therefore we predict that power bias correction will not be effective when noise dominates over the connectivity processes.

On top of the power bias itself, noise correlations R_{noise} contribute additively to functional connectivity estimates but their contribution is dampened at high SNR (second term in Eq. 5). Our strategy to model background noise in MEG data enforces that noise correlations vanish, so in theory $R_{\text{noise}} = 0$ (see defining property i below). In the context of MEG signals, persisting but spurious linear noise correlations are still bound to emerge in practice from linear mixing associated with source projection, although we can expect spatial leakage correction to dampen these correlations. So we can expect *a priori* that the power bias remains the dominating feature over noise correlations. Their effect can be eliminated by mere subtraction (see numerator in Eq. 6) alongside the power bias renormalization *per se*.

Modeling background noise. To make the correction procedure (6) applicable in practice, the SNR-dependent renormalization factor (as well as the noise correlation subtractive term) must be expressible in terms of parameters that are accessible to experimental data. We thus avoided formulating Eqs. (5) and (6) in terms of, e.g., σ_{x_0} and σ_{y_0} since the connectivity processes x_0 and y_0 are unknown. The noise processes $\epsilon_x(t)$ and $\epsilon_y(t)$ are also inaccessible, but second-order statistics (variances $\sigma_{\epsilon_x}^2$ and $\sigma_{\epsilon_y}^2$ appearing through the SNR parameters, Eq. 4, and correlation R_{noise}) are necessary. That is why our mathematical model must be supplemented with an explicit neurobiological model that isolates background noise processes and enables estimation of their statistics. Our broad strategy is as follows:

(i) We define a model of ϵ_x and ϵ_y from signals devoid of the neural couplings of interest. In our analyses, we used either the "cerebral background noise" constructed from signal periods of coincident non-bursting activity in resting-state MEG recordings, or the "measurement noise" from empty-room MEG recordings (see main text). These model signals are not simultaneous with the experimental signals x and y, but they can be used nonetheless to estimate the required statistics. Note in particular that consistency of the model noises requires that their functional connectivity theoretically vanishes; e.g., we should expect $R_{\text{noise}} \approx 0$ when investigating linear correlations (modulo spatial leakage effects).

(ii) We further assume that the background noise processes ϵ_x, ϵ_y contribute linearly to the experimental signals (2), and that they are independent of the neural connectivity processes x_0, y_0. This cannot be verified experimentally since by design the model signals are not simultaneous to the recordings x, y, although this is obviously true for measurement noise. For cerebral background noise, this amounts to genuinely assume that neural processes generating functional connectivity (e.g., oscillatory bursts; Seedat et al., 2020) and neural processes that do not participate to functional connectivity (e.g., non-bursting oscillations; see main text) are temporally independent.

Application to phase connectivity. It is noteworthy that the above considerations directly apply to the neuro-scientifically relevant phase connectivity measure known as coherence (Halliday et al., 1995), as it merely corresponds to a spectrally-resolved version of linear correlation. In this framework, results (5) and (6) hold with correlations (R and R_{noise}) replaced by complex-valued coherency estimates and signal variances (σ_x^2, σ_y^2, $\sigma_{\epsilon_x}^2$, and $\sigma_{\epsilon_y}^2$ appearing in Eq. 4), by power spectral densities. The generalization to other phase connectivity measures such as the phase-locking value turns out to be much more challenging to handle. We do not pursue this here and consider in detail the case of amplitude connectivity relevant to the electrophysiological mapping of intrinsic brain networks.

Mathematical analysis: Derivation of Eq. (5). We start by expanding the numerator in the right-hand side of Eq. (1),

$$\begin{aligned}\text{cov}(x, y) &= \text{cov}\,(x_0, y_0) + \text{cov}(x_0, \epsilon_y) \\ &\quad + \text{cov}\,(\epsilon_x, y_0) + \text{cov}(\epsilon_x, \epsilon_y)\,.\end{aligned} \tag{7}$$

The two middle terms must vanish since connectivity processes x_0, y_0 and background noises ϵ_x, ϵ_y are assumed to be independent, leaving $\text{cov}(x, y) = \text{cov}\,(x_0, y_0) + \text{cov}(\epsilon_x, \epsilon_y)$. Similarly, in the denominator

$$\sigma_x^2 = \sigma_{x_0}^2 + 2\text{cov}\,(x_0, \epsilon_x) + \sigma_{\epsilon_x}^2 = \sigma_{x_0}^2 + \sigma_{\epsilon_x}^2\,. \tag{8}$$

Combined with our definition (4) of the SNR, this equation can be recast as

$$\frac{1}{\sigma_x^2} = \frac{1}{\sigma_x^2}\left(\frac{\sigma_x^2 - \sigma_{\epsilon_x}^2}{\sigma_{x_0}^2}\right) = \frac{1 - \frac{\sigma_{\epsilon_x}^2}{\sigma_x^2}}{\sigma_{x_0}^2} = \frac{1 - \text{SNR}_x^{-2}}{\sigma_{x_0}^2}\,, \tag{9}$$

the factor between parentheses being equal to one. A similar equation holds for the signal y. Plugging these results back into Eq. (1) and using Eq. (4) once more leads to the sought Eq. (5).

Appendix A.2. Amplitude correlation

Setup. Assessing the simultaneity of the rise and fall of transient neural rhythms in two brain areas requires first to isolate the amplitude time course of these rhythms and then to assess their temporal covariation. Isolation of rhythmic activity itself

in MEG signals can be performed by band-pass filtering, so we will assume hereafter that x and y correspond to sufficiently narrow-band signals. In this context, we may interpret the signals x_0 and y_0 in Eq. (2) as local neural rhythmic activity that generates their functional coupling, and ϵ_x and ϵ_y as the band-filtered part of the rest of local neural activity not involved in this functional coupling (possibly wideband, though our results suggest that they contain sustained non-bursting oscillations; see main text) along with measurement noise.

Time-varying oscillatory amplitudes can then be conveniently extracted using the Hilbert transform, which factorizes any band-limited signal, say $x(t)$, as $A_x(t)\, \mathrm{e}^{\mathrm{i}\phi_x(t)}$, where A_x denotes its amplitude time course (a.k.a. Hilbert envelope) and ϕ_x its instantaneous phase time course. This factorization applies to both connectivity and noise processes, so the relation $x = x_0 + \epsilon_x$ becomes

$$A_x \mathrm{e}^{\mathrm{i}\phi_x} = A_{x_0} \mathrm{e}^{\mathrm{i}\phi_{x_0}} + A_{\epsilon_x} \mathrm{e}^{\mathrm{i}\phi_{\epsilon_x}} \tag{10}$$

and similarly for the signal y. The mathematics of Hilbert transformation needed to develop our theory is briefly reviewed below.

In this framework, the co-occurrence of transient brain rhythms in two brain signals x and y translates into the temporal dependence of their two oscillatory amplitudes A_x and A_y, which is conventionally measured as their correlation, i.e., $\mathrm{AEC} = \mathrm{corr}[A_x, A_y]$. Our goal is to express this functional connectivity measure in terms of the neural $\mathrm{AEC}_0 = \mathrm{corr}[A_{x_0}, A_{y_0}]$ involving only oscillatory amplitudes A_{x_0} and A_{y_0} generating the coupling, and of a version of the SNR pertaining to oscillatory amplitudes that will be defined below.

Amplitude vs. power correlation. The major technical difficulty in this endeavor is that A_x is a nonlinear function of A_{x_0} (see Eq. 30 below), preventing us to follow the strategy used in Appendix A.1 for linear correlation. Fortunately, it turns out that the correlation of amplitudes in the AEC can be replaced, to an extremely good approximation, by the correlation of amplitudes squared (Wens, 2015), so

$$\mathrm{AEC} \approx \mathrm{corr}\left[A_x^2, A_y^2\right] = \frac{\mathrm{cov}\left(A_x^2, A_y^2\right)}{\sigma_{A_x^2}\, \sigma_{A_x^2}} \tag{11}$$

and similarly for AEC_0. The correlation of amplitudes squared is actually closely related to the band-limited power correlation used by de Pasquale and colleagues (de Pasquale et al., 2010; Della Penna et al., 2019), which has been observed not only to yield the same functional brain networks than AEC but also quantitatively very similar functional connectivity estimates (Sjogard et al., 2019). The mathematical derivation of the approximation (11) is derived in Appendix E of Wens (2015). It is noteworthy that this approximation could have been avoided altogether, had we measured intrinsic functional connectivity with power correlation from the start; we did not do so here to keep in line with a large portion of the literature on MEG amplitude connectivity.

Power bias in amplitude correlation. Using Eq. (11) as starting point, we show below how to generalize Eq. (5) for linear correlations to the case of narrow-band AEC. The main result of this analysis reads

$$\begin{aligned}\mathrm{AEC} \approx \mathrm{AEC}_0 &\sqrt{1-\left(\mathrm{SNR}_x^{\mathrm{ampl}}\right)^{-2}}\ \sqrt{1-\left(\mathrm{SNR}_y^{\mathrm{ampl}}\right)^{-2}} \\ &+ \frac{\mathrm{cov}\left(A_{\epsilon_x}^2, A_{\epsilon_y}^2\right)}{\sigma_{A_x^2}\sigma_{A_y^2}} + \frac{\mathscr{L}(x,y) + \mathscr{L}(x, \mathsf{H}y)}{\sigma_{A_x^2}\sigma_{A_y^2}}\,.\end{aligned} \tag{12}$$

The first term embodies the power bias in AEC. It is formally similar to that in linear correlation (Eq. 5) except that it involves an amplitude-specific version of the SNR defined as

$$\mathrm{SNR}_x^{\mathrm{ampl}} = \frac{\sigma_{A_x^2}}{\sqrt{\sigma_{A_{\epsilon_x}^2}^2 + 2\langle A_x^2\rangle\langle A_{\epsilon_x}^2\rangle - 2\langle A_{\epsilon_x}^2\rangle^2}} \tag{13}$$

and similarly for A_y. To get some intuition on this unusual SNR measure, we plot in Fig. S1 this quantity $\mathrm{SNR}_x^{\mathrm{ampl}}$ as a function of the standard SNR_x (Eq. 4) for the synthetic electrophysiological signals used to explore our theory numerically (see main text). In this case, the relation appeared linear, with a slope estimated as $0.71 = 1/\sqrt{2}$ by linear regression, so the nonlinearity factor $\sqrt{1-\left(\mathrm{SNR}_x^{\mathrm{ampl}}\right)^{-2}}$ may be simplified to $\sqrt{1-2\,(\mathrm{SNR}_x)^{-2}}$ (see caption of Fig. 1). It turns out that this simplification emerged in this plot only because our synthetic signals were close to being gaussian (see the last paragraph of this Appendix) but it does not hold in general for non-gaussian signals such as experimental MEG recordings. For that reason, the predicted power bias effect sizes reported in Fig. 1(c) expressed in terms of the "gaussian" SNR may not be directly interpreted in terms of the SNR of MEG signals. Since Eq. (12) demonstrates that the power bias in AEC only depends on the SNR through the amplitude-specific version (13), the correct way to read off predictions from Fig. 1(c) is to estimate $\mathrm{SNR}_x^{\mathrm{ampl}}$ from experimental MEG recordings, artificially convert them into "gaussian" SNR according to $\mathrm{SNR}_x^{\mathrm{gaussian}} = \sqrt{2}\ \mathrm{SNR}_x^{\mathrm{ampl}}$ (Fig. S1), and use these values in Fig. 1(c).

The second term in Eq. (12) represents the spurious inflating effect of noise amplitude correlations on AEC, in complete analogy with the contribution of R_{noise} to linear correlation (Eq. 5). See SI Results (Appendix C) for an illustration of this effect in synthetic data. In principle, this term should vanish since a good model of "background noise" must be devoid of any amplitude coupling (see part i of noise model definition in section A.1), i.e., $\mathrm{cov}(A_{\epsilon_x}^2, A_{\epsilon_y}^2) \approx 0$. This was verified explicitly in the case of cerebral background noise modeled as non-bursting brain activity (see main text). So this effect only depends on noise linear correlations and is likely subdominant after leakage correction, but we nevertheless kept track of it for the sake of generality (see discussion in section A.1 below Eq. 6).

The third and last term involves the quantity

$$\mathscr{L}(x,y) = 8\left[\mathrm{cov}(x,y)\,\mathrm{cov}(\epsilon_x,\epsilon_y) - \mathrm{cov}(\epsilon_x,\epsilon_y)^2\right] \tag{14}$$

depending on the linear covariances between MEG signals x, y and between the corresponding noise signals ϵ_x, ϵ_y, along

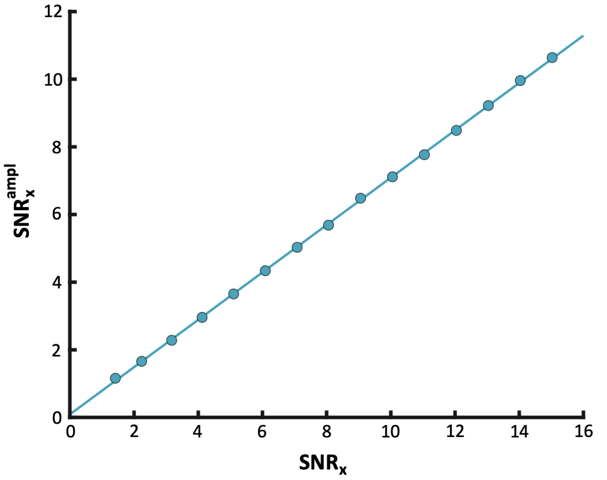

Figure S1: *Linear relationship between the amplitude-specific SNR (Eq. 13) and the classical SNR (Eq. 4) for near-gaussian synthetic MEG signals (see main text).* The regression model $\mathrm{SNR}_x^{\mathrm{ampl}} = \mathrm{SNR}/\sqrt{2}$ is superimposed (see last paragraph of this Appendix for the complete mathematical relationship).

with a similar contribution $\mathscr{L}(x, \mathsf{H}y)$ that involves the Hilbert transforms $\mathsf{H}y$ and $\mathsf{H}\epsilon_y$ (see Eq. 18 below). This last contribution is proportional to the noise correlation R_{noise} and is thus likely subdominant after leakage correction. As we show below, $\mathscr{L}(x, y)$ is also proportional to the neural zero phase-lag coupling R_0 (and $\mathscr{L}(x, \mathsf{H}y)$ to the neural coupling at $\pi/2$ phase lag; see Eq. 28). Although R_0 is generally thought to vanish in the resting state, Sjogard et al. (2019) suggested the existence of spontaneous quasi-zero lag correlations, at least within the default-mode network. For this reason, we will remain general and retain all contributions in Eq. (12).

Let us finally comment on the approximative nature of our result (12). It originates from two approximations needed to carry out analytical developments: Eq. (11) and deviations from the narrow-band limit (necessary to rely on the machinery of the Hilbert transform, see mathematical derivations below). Our validations with synthetic data (Fig. 1d) actually establish the excellent accuracy of both approximations, so that for all practical purposes the approximation sign ($\approx$) may thus be replaced by an equality in Eq. (12).

Power bias correction by renormalization. The general discussion that follows Eq. (5) holds without change here, and in particular the neural amplitude correlation AEC_0 can be recovered via a renormalization procedure

$$\mathrm{AEC}_{\mathrm{ren}} = \frac{\mathrm{AEC} - \frac{\mathrm{cov}\left(A_{\epsilon_x}^2, A_{\epsilon_y}^2\right)}{\sigma_{A_x^2}\sigma_{A_y^2}} - \frac{\mathscr{L}(x,y)+\mathscr{L}(x,\mathsf{H}y)}{\sigma_{A_x^2}\sigma_{A_y^2}}}{\sqrt{1-\left(\mathrm{SNR}_x^{\mathrm{ampl}}\right)^{-2}}\ \sqrt{1-\left(\mathrm{SNR}_y^{\mathrm{ampl}}\right)^{-2}}}\,. \qquad (15)$$

Critically, this quantity is expressed in terms of parameters that are experimentally accessible to MEG recordings. The two subtractive terms in the numerator correct for possible (and likely subdominant) noise correlations and zero phase-lag coupling, and the denominator corrects for the power bias itself. The estimation of second- and fourth-order statistics of the noise signals ϵ_x and ϵ_y relies once again on a separate modeling step of background noise. See the related discussion in Appendix A.1. Analogously to the case of linear correlation, good conditioning of the renormalization (15) requires that both $\mathrm{SNR}_x^{\mathrm{ampl}}$ and $\mathrm{SNR}_y^{\mathrm{ampl}}$ exceed 1.

We now turn to the mathematical justification of our main theoretical result (12).

Mathematical derivations: Separation of timescales in oscillatory dynamics. We consider a general signal $x(t)$, whose Fourier transform $\widehat{x}$ is supported on a frequency band of center ν_0 and half-width $\Delta\nu < \nu_0$. Such signal can be factorized according to

$$x(t) = A_x(t)\cos\phi_x(t)\,, \qquad (16)$$

where the amplitude A_x and phase ϕ_x are determined through the relationship

$$A_x(t)\,\mathrm{e}^{\mathrm{i}\phi_x(t)} = 2\int_{\nu_0-\Delta\nu}^{\nu_0+\Delta\nu} \widehat{x}(\nu)\,\mathrm{e}^{2\pi\mathrm{i}\nu t}\,\mathrm{d}\nu\,. \qquad (17)$$

The Hilbert transform $\mathsf{H}x$ corresponds to the signal (16) but with 90-degree lagged phase $\phi_x(t) - \pi/2$, i.e.,

$$\mathsf{H}x(t) = A_x(t)\sin\phi_x(t)\,. \qquad (18)$$

In the narrow-band limit $\Delta\nu \ll \nu_0$ pertaining to oscillatory dynamics, A_x captures slow modulations of the amplitude and ϕ_x rapid phase oscillations at an instantaneous frequency that varies slowly around the center frequency ν_0. A succinct way to understand this basic property is to apply a change of variable $\nu \longrightarrow \nu_0 + \nu$ in the Fourier integral (17), which yields

$$A_x(t)\,\mathrm{e}^{\mathrm{i}\phi_x(t)} = \mathrm{e}^{2\pi\mathrm{i}\nu_0 t} \times \left[2\int_{-\Delta\nu}^{\Delta\nu} \widehat{x}(\nu_0+\nu)\,\mathrm{e}^{2\pi\mathrm{i}\nu t}\,\mathrm{d}\nu\right]. \qquad (19)$$

The factor between brackets in the right-hand side determines a complex-valued signal $A_x(t)\,\mathrm{e}^{\mathrm{i}\delta_x(t)}$ with same amplitude than x, a phase $\delta_x = \phi_x - 2\pi\nu_0 t$ that corresponds to the phase shift between x and the carrying oscillation at frequency ν_0, and a Fourier spectrum restricted to frequencies below $\Delta\nu$. This implies that the amplitude A_x and the phase shift δ_x both evolve on timescales much slower than the carrying oscillation.

Mathematical derivations: Technical identities on time averages. This separation of timescales applies to any narrow-band signal; in our setup x may be any one of the connectivity processes x_0, y_0 or background noises ϵ_x, ϵ_y. Together with the assumption that connectivity processes and noise are temporally independent (see noise model assumption ii in Appendix A.1), this is key to estimate analytically the several time averages that we will encounter below. The main results of this section are Eqs. (20), (24), and (29). They are presented here as approximate in the sense that they work in the narrow-band limit $\Delta\nu \ll \nu_0$, but this condition holds to a good approximation in our spectrally resolved MEG data (see main text) so in practice the approximation errors are small. Of note, no hypothesis on the statistical (in)dependence of amplitudes and phases will be required here.

While evaluating the correlation of amplitudes squared (11), a number of simplifications emerge from identities of the form

$$\left\langle A_{x_0}^a A_{\epsilon_x}^b A_{y_0}^c A_{\epsilon_y}^d \cos\left(\phi_{x_0} - \phi_{\epsilon_x}\right)\right\rangle \approx 0 \tag{20}$$

holding for any non-negative integers $a, b, c, d \geq 0$, and similar identities involving the phase lag $\phi_{y_0} - \phi_{\epsilon_y}$. To demonstrate Eq. (20), we expand the left-hand side with a classical trigonometric identity and apply the independence of x_0, y_0 and ϵ_x, ϵ_y to factorize time averages to obtain

$$\begin{aligned}&\langle A_{x_0}^a A_{y_0}^c \cos\phi_{x_0}\rangle\langle A_{\epsilon_x}^b A_{\epsilon_y}^d \cos\phi_{\epsilon_x}\rangle \\ &+ \langle A_{x_0}^a A_{y_0}^c \sin\phi_{x_0}\rangle\langle A_{\epsilon_x}^b A_{\epsilon_y}^d \sin\phi_{\epsilon_x}\rangle\,.\end{aligned} \tag{21}$$

Each factor in this expression vanishes approximately because amplitudes remain approximately constant over any cycle of the phase oscillations. Let us consider for instance the first factor; inserting $\phi_{x_0} = 2\pi\nu_0 t + \delta_{x_0}$ yields

$$\begin{aligned}\langle A_{x_0}^a A_{y_0}^c \cos\phi_{x_0}\rangle &= \langle A_{x_0}^a A_{y_0}^c \cos\delta_{x_0} \cos\left(2\pi\nu_0 t\right)\rangle \\ &- \langle A_{x_0}^a A_{y_0}^c \sin\delta_{x_0} \sin\left(2\pi\nu_0 t\right)\rangle\,.\end{aligned} \tag{22}$$

The Riemann-Lebesgue lemma establishes that each of these two terms vanish in the limit $\nu_0/\Delta\nu \to \infty$, but this formal argument can be explained on the basis of the separation of timescales. Slow amplitude and phase modulations involve frequencies below $\Delta\nu \ll \nu_0$ and so they are fairly constant over short time periods corresponding to one cycle of the rapid carrying oscillation at frequency ν_0. This allows to factorize time averages into fast oscillatory averages over one cycle and slow modulatory averages over long times, e.g.,

$$\begin{aligned}\langle A_{x_0}^a A_{y_0}^c \cos\phi_{x_0}\rangle &\approx \langle A_{x_0}^a A_{y_0}^c \cos\delta_{x_0}\rangle\langle \cos\left(2\pi\nu_0 t\right)\rangle \\ &- \langle A_{x_0}^a A_{y_0}^c \sin\delta_{x_0}\rangle\langle \sin\left(2\pi\nu_0 t\right)\rangle\,.\end{aligned} \tag{23}$$

This expression vanishes since $\langle\cos\left(2\pi\nu_0 t\right)\rangle = \langle\sin\left(2\pi\nu_0 t\right)\rangle = 0$ over any cycle. The same holds for all terms encountered above. This ends the demonstration of the identities (20).

We will also encounter non-vanishing averages involving second powers of cosines. First,

$$\left\langle A_{x_0}^2 A_{\epsilon_x}^2 \cos\left(\phi_{x_0} - \phi_{\epsilon_x}\right)^2\right\rangle \approx \frac{\langle A_{x_0}^2\rangle\langle A_{\epsilon_x}^2\rangle}{2} \tag{24}$$

and likewise for the y signals. To derive Eq. (24), we again use trigonometry to rewrite the left-hand side as $\langle A_{x_0}^2 A_{\epsilon_x}^2 \left[1 + \cos 2\left(\phi_{x_0} - \phi_{\epsilon_x}\right)\right]/2\rangle$. The cosine term vanishes in the narrow-band approximation for the exact same reasons than Eq. (20) holds, leaving $\langle A_{x_0}^2 A_{\epsilon_x}^2\rangle/2$. The latter average factorizes since connectivity processes and noise are independent, leading us back to Eq. (24).

The second non-vanishing identity is

$$\begin{aligned}&\left\langle A_{x_0} A_{\epsilon_x} A_{y_0} A_{\epsilon_y} \cos\left(\phi_{x_0} - \phi_{\epsilon_x}\right)\cos(\phi_{y_0} - \phi_{\epsilon_y})\right\rangle \\ &\qquad \approx 2\langle x_0 y_0\rangle\langle\epsilon_x\epsilon_y\rangle + 2\langle x_0 \mathsf{H} y_0\rangle\langle\epsilon_x \mathsf{H}\epsilon_y\rangle\,.\end{aligned} \tag{25}$$

To establish this, we start by expanding both cosines as we did below Eq. (20). Recognizing the signals x_0, ϵ_x and their Hilbert transform through Eqs. (16) and (18) allows to write

$$\begin{aligned}&A_{x_0} A_{\epsilon_x} \cos\left(\phi_{x_0} - \phi_{\epsilon_x}\right) \\ &\quad = A_{x_0}\cos\phi_{x_0} A_{\epsilon_x}\cos\phi_{\epsilon_x} + A_{x_0}\sin\phi_{x_0} A_{\epsilon_x}\sin\phi_{\epsilon_x} \\ &\quad = x_0\epsilon_x + \mathsf{H}x_0\mathsf{H}\epsilon_x\end{aligned}$$

and similarly for the y signals. Inserting these relationships into the left-hand side of Eq. (25) yields a time average $\langle(x_0\epsilon_x + \mathsf{H}x_0\mathsf{H}\epsilon_x)\,(y_0\epsilon_y + \mathsf{H}y_0\mathsf{H}\epsilon_y)\rangle$. Expanding the product and using the independence of x_0, y_0 and ϵ_x, ϵ_y, we obtain

$$\begin{aligned}&\langle x_0 y_0\rangle\langle\epsilon_x\epsilon_y\rangle + \langle x_0\mathsf{H}y_0\rangle\langle\epsilon_x\mathsf{H}\epsilon_y\rangle \\ &\quad + \langle\mathsf{H}x_0 y_0\rangle\langle\mathsf{H}\epsilon_x\epsilon_y\rangle + \langle\mathsf{H}x_0\mathsf{H}y_0\rangle\langle\mathsf{H}\epsilon_x\mathsf{H}\epsilon_y\rangle\,.\end{aligned}$$

It turns out that the first and last terms are equal to each other, and likewise for the second and third terms, from which Eq. (25) follows. For example,

$$\begin{aligned}\langle x_0 y_0\rangle &= \left\langle A_{x_0} A_{y_0}\cos\phi_{x_0}\cos\phi_{y_0}\right\rangle \\ &= \left\langle A_{x_0} A_{y_0}\frac{\cos(\phi_{x_0} + \phi_{y_0}) + \cos(\phi_{x_0} - \phi_{y_0})}{2}\right\rangle\end{aligned} \tag{26}$$

and

$$\begin{aligned}\langle\mathsf{H}x_0\mathsf{H}y_0\rangle &= \left\langle A_{x_0} A_{y_0}\sin\phi_{x_0}\sin\phi_{y_0}\right\rangle \\ &= \left\langle A_{x_0} A_{y_0}\frac{-\cos\left(\phi_{x_0} + \phi_{y_0}\right) + \cos\left(\phi_{x_0} - \phi_{y_0}\right)}{2}\right\rangle\end{aligned} \tag{27}$$

both converge to the same value $\langle A_{x_0}A_{y_0}\cos(\delta_{x_0} - \delta_{y_0})\rangle/2$ because the time average containing the high-frequency oscillation $\cos(\phi_{x_0} + \phi_{y_0}) = \cos(4\pi\nu_0 t + \delta_{x_0} + \delta_{y_0})$ vanishes in the narrow-band approximation once again thanks to the separation of timescales.

It is noteworthy that the first term in the right-hand side of Eq. (25) is closely related to the quantity $\mathscr{L}(x, y)$ defined in Eq. (14). More specifically,

$$\mathscr{L}(x, y) = 8\,\mathrm{cov}(x_0, y_0)\,\mathrm{cov}(\epsilon_x, \epsilon_y) = 8\,\langle x_0 y_0\rangle\,\langle\epsilon_x\epsilon_y\rangle\,. \tag{28}$$

The first equality follows by inserting the relationship (2) between connectivity processes and measured signals into the definition (14) and using the independence of connectivity and noise processes to set both $\mathrm{cov}(x_0, \epsilon_y)$ and $\mathrm{cov}(\epsilon_x, y_0)$ to zero. The second equality follows from the definition of covariance (reviewed right below Eq. 1) and the fact that all signal averages $\langle x_0\rangle$, $\langle y_0\rangle$, $\langle\epsilon_x\rangle$, and $\langle\epsilon_y\rangle$ vanish (which are particular cases of Eq. 20). Identity (25) can then be recast as

$$\begin{aligned}&\left\langle A_{x_0} A_{\epsilon_x} A_{y_0} A_{\epsilon_y}\cos\left(\phi_{x_0} - \phi_{\epsilon_x}\right)\cos\left(\phi_{y_0} - \phi_{\epsilon_y}\right)\right\rangle \\ &\qquad \approx \frac{\mathscr{L}(x, y) + \mathscr{L}(x, \mathsf{H}y)}{4}\,.\end{aligned} \tag{29}$$

The significance of Eq. (28) is that it establishes the claim made below Eq. (14) that $\mathscr{L}(x, y)$ is proportional to the neural zero-lag correlation R_0. Similarly, by definition (18) of the Hilbert transform, $\mathscr{L}(x, \mathsf{H}y)$ is proportional to a measure $\mathrm{corr}[x_0, \mathsf{H}y_0]$ of neural 90-degree phase lag coupling.

Mathematical derivations: Proof of Eq. (12). After these technical preliminaries, we are now in position to derive our main theoretical result (12) from Eq. (11). Our starting point is the modulus squared of Eq. (10),

$$A_x^2 = A_{x_0}^2 + A_{\epsilon_x}^2 + 2A_{x_0}A_{\epsilon_x}\cos\left(\phi_{x_0} - \phi_{\epsilon_x}\right) . \qquad (30)$$

A similar relation holds for the signal y. These expressions play the same role here than Eq. (2) did in Appendix A.1, but the situation is somewhat more intricate due to the interplay between amplitude and phase-lag dynamics evidenced by the rightmost term in Eq. (30).

It is slightly simpler to deal with the denominator of Eq. (11) first. We insert the decomposition (30) into the defining expression of the variance of amplitude squared $\sigma^2_{A_x^2} = \mathrm{cov}(A_x^2, A_x^2)$. Expanding and gathering terms where possible, we find that $\sigma^2_{A_x^2}$ equals to

$$\begin{aligned}\sigma^2_{A_{x_0}^2} + \sigma^2_{A_{\epsilon_x}^2} &+ 2\mathrm{cov}(A_{x_0}^2, A_{\epsilon_x}^2) + 4\langle A_{x_0}^2 A_{\epsilon_x}^2 \cos(\phi_{x_0} - \phi_{\epsilon_x})^2\rangle \\ &- 4\left\langle A_{x_0}A_{\epsilon_x}\cos(\phi_{x_0} - \phi_{\epsilon_x})\right\rangle^2 \\ &+ 2\langle (A_{x_0}^2 + A_{\epsilon_x}^2)A_{x_0}A_{\epsilon_x}\cos(\phi_{x_0} - \phi_{\epsilon_x})\rangle \\ &- 2\langle A_{x_0}^2 + A_{\epsilon_x}^2\rangle\langle A_{x_0}A_{\epsilon_x}\cos(\phi_{x_0} - \phi_{\epsilon_x})\rangle .\end{aligned}$$

This daunting expression simplifies in the narrow-band limit, as the third term vanishes due to the independence of connectivity and noise processes (see model noise hypothesis ii in Appendix A.1) and so do the fifth to last terms thanks to identities (20). Our result (24) takes care of the fourth term and leads to

$$\sigma^2_{A_x^2} \approx \sigma^2_{A_{x_0}^2} + \sigma^2_{A_{\epsilon_x}^2} + 2\langle A_{x_0}^2\rangle\langle A_{\epsilon_x}^2\rangle . \qquad (31)$$

We then take the time average of the decomposition formula (30) and apply an identity (20) to simplify the last term,

$$\begin{aligned}\langle A_x^2\rangle &\approx \langle A_{x_0}^2\rangle + \langle A_{\epsilon_x}^2\rangle + 2\langle A_{x_0}A_{\epsilon_x}\cos(\phi_{x_0} - \phi_{\epsilon_x})\rangle \\ &\approx \langle A_{x_0}^2\rangle + \langle A_{\epsilon_x}^2\rangle .\end{aligned} \qquad (32)$$

Combining the two preceding equations, we obtain

$$\begin{aligned}\sigma^2_{A_x^2} &\approx \sigma^2_{A_{x_0}^2} + \sigma^2_{A_{\epsilon_x}^2} + 2\left(\langle A_x^2\rangle - \langle A_{\epsilon_x}^2\rangle\right)\langle A_{\epsilon_x}^2\rangle \\ &= \sigma^2_{A_{x_0}^2} + \sigma^2_{A_{\epsilon_x}^2} + 2\langle A_x^2\rangle\langle A_{\epsilon_x}^2\rangle - 2\langle A_{\epsilon_x}^2\rangle^2 .\end{aligned} \qquad (33)$$

This is the analog of the intermediate result $\sigma_x^2 = \sigma_{x_0}^2 + \sigma_{\epsilon_x}^2$ of Appendix A.1 but for amplitude squared. When placed in the denominator, we can recast Eq. (33) as

$$\begin{aligned}\frac{1}{\sigma^2_{A_x^2}} &\approx \frac{1}{\sigma^2_{A_x^2}}\left(\frac{\sigma^2_{A_x^2} - \sigma^2_{A_{\epsilon_x}^2} - 2\langle A_x^2\rangle\langle A_{\epsilon_x}^2\rangle + 2\langle A_{\epsilon_x}^2\rangle^2}{\sigma^2_{A_{x_0}^2}}\right) \\ &= \frac{1}{\sigma^2_{A_{x_0}^2}}\left(1 - \frac{\sigma^2_{A_{\epsilon_x}^2} + 2\langle A_x^2\rangle\langle A_{\epsilon_x}^2\rangle - 2\langle A_{\epsilon_x}^2\rangle^2}{\sigma^2_{A_x^2}}\right) .\end{aligned} \qquad (34)$$

Comparison with the analogous result $1/\sigma_x^2 = (1 - \mathrm{SNR}_x^{-2})/\sigma_{x_0}^2$ at the end of Appendix A.1 motivates our definition of the amplitude-specific SNR measure (13), since then

$$\frac{1}{\sigma^2_{A_x^2}} \approx \frac{1 - \left(\mathrm{SNR}_x^{\mathrm{ampl}}\right)^{-2}}{\sigma^2_{A_{x_0}^2}} . \qquad (35)$$

A similar result holds for the signal y. This handles the case of the denominator in Eq. (11); we now turn to its numerator.

We need to find the analog of the intermediate result $\mathrm{cov}(x, y) = \mathrm{cov}\,(x_0, y_0) + \mathrm{cov}(\epsilon_x, \epsilon_y\,)$ of Appendix A.1. Expanding the covariance $\mathrm{cov}(A_x^2, A_y^2)$ with the decomposition formula (30) and gathering terms leads to

$$\begin{aligned}\mathrm{cov}(A_{x_0}^2, A_{y_0}^2) &+ \mathrm{cov}(A_{x_0}^2, A_{\epsilon_y}^2) + \mathrm{cov}(A_{\epsilon_x}^2, A_{y_0}^2) + \mathrm{cov}(A_{\epsilon_x}^2, A_{\epsilon_y}^2) \\ &+ 2\mathrm{cov}\left(A_{x_0}^2 + A_{\epsilon_x}^2, A_{y_0}A_{\epsilon_y}\cos(\phi_{y_0} - \phi_{\epsilon_y})\right) \\ &+ 2\mathrm{cov}\left(A_{x_0}A_{\epsilon_x}\cos(\phi_{x_0} - \phi_{\epsilon_x}), A_{y_0}^2 + A_{\epsilon_y}^2\right) \\ &+ 4\mathrm{cov}\left(A_{x_0}A_{\epsilon_x}\cos(\phi_{x_0} - \phi_{\epsilon_x}), A_{y_0}A_{\epsilon_y}\cos(\phi_{y_0} - \phi_{\epsilon_y})\right) .\end{aligned}$$

Again, several simplifications take place in the narrow-band limit. The second and third terms vanish according to the independence of connectivity and noise processes (see model noise hypothesis ii in Appendix A.1); the covariances in the fifth and sixth terms too since they involve averages of the form (20). The last term also simplifies to

$$4\left\langle A_{x_0}A_{\epsilon_x}A_{y_0}A_{\epsilon_y}\cos\left(\phi_{x_0} - \phi_{\epsilon_x}\right)\cos(\phi_{y_0} - \phi_{\epsilon_y})\right\rangle$$

again thanks to Eq. (20), and thus it equals to $\mathscr{L}(x, y) + \mathscr{L}(x, Hy)$ by our last identity (29). Gathering these observations together, we find

$$\begin{aligned}\mathrm{cov}\left(A_x^2, A_y^2\right) &\approx \mathrm{cov}\left(A_{x_0}^2, A_{y_0}^2\right) + \mathrm{cov}\left(A_{\epsilon_x}^2, A_{\epsilon_y}^2\right) \\ &\quad + \mathscr{L}(x, y) + \mathscr{L}(x, \mathrm{H}y) .\end{aligned} \qquad (36)$$

Our main result is now at hand. Equation (11) for the AEC consists in normalizing the covariance (36) of amplitudes squared by their standard deviation, which using Eq. (33) yields Eq. (12).

Mathematical derivations: Amplitude-specific SNR for gaussian signals. For completeness, we explore here the link between the amplitude-specific $\mathrm{SNR}_x^{\mathrm{ampl}}$ and the standard SNR_x in the case of gaussian signals, and thereby explain Fig. S1 analytically. We start from the observation that definition (13) involves a combination of averages of amplitude squared $\langle A_x^2\rangle$, $\langle A_{\epsilon_x}^2\rangle$ and to the fourth power $\langle A_x^4\rangle$, $\langle A_{\epsilon_x}^4\rangle$, which can be expressed in terms of variance $\sigma_x^2 = \langle (x - \langle x\rangle)^2\rangle$ and fourth-order moment $\mu_x^4 = \langle (x - \langle x\rangle)^4\rangle$ according to

$$\langle A_x^2\rangle \approx 2\sigma_x^2 \qquad \text{and} \qquad \langle A_x^4\rangle \approx 8\mu_x^4/3 , \qquad (37)$$

and similarly for ϵ_x. These identities follow in the narrow-band limit from arguments very similar to above. Briefly, applying Eq. (16), the zero-mean property $\langle x\rangle = 0$, and trigonometric identities yields

$$\sigma_x^2 = \left\langle A_x^2\,\cos^2(\phi_x)\right\rangle = \frac{\langle A_x^2\rangle + \langle A_x^2\cos(2\phi_x)\rangle}{2} \approx \frac{\langle A_x^2\rangle}{2} \qquad (38)$$

and

$$\mu_x^4 = \frac{3\langle A_x^4\rangle}{8} + \frac{\langle A_x^4\cos(2\phi_x)\rangle}{2} + \frac{\langle A_x^4\cos(4\phi_x)\rangle}{8} \approx \frac{3\langle A_x^4\rangle}{8} , \qquad (39)$$

with the last equalities holding in the narrow-band limit.

Equations (37) allow to recast the definition (13) of the amplitude-specific SNR as

$$\mathrm{SNR}_x^{\mathrm{ampl}} \approx \sqrt{\frac{8\mu_x^4/3 - 4\sigma_x^4}{8\mu_{\epsilon_x}^4/3 - 12\sigma_{\epsilon_x}^4 + 8\sigma_x^2\sigma_{\epsilon_x}^2}}\,. \tag{40}$$

So far this result holds for arbitrary narrow-band signals, but it simplifies a great deal in the case of gaussian signals because they obey the zero-kurtosis identity $\mu_x^4 = 3\sigma_x^4$. With the help of Eq. (4), we find

$$\mathrm{SNR}_x^{\mathrm{ampl}} \approx \sqrt{\frac{\sigma_x^4}{2\sigma_x^2\sigma_{\epsilon_x}^2 - \sigma_{\epsilon_x}^4}} = \frac{\mathrm{SNR}_x}{\sqrt{2 - \mathrm{SNR}_x^{-2}}}\,, \tag{41}$$

which comes close to $\mathrm{SNR}_x^{\mathrm{ampl}} = \mathrm{SNR}_x/\sqrt{2}$ (except for SNR_x values very close to 1), in line with Fig. S1.

Appendix B. SI Methods

Appendix B.1. More on background noise models

Our procedure to model cerebral background noise allows to access noise statistics but not its full time course synchronized with experimental MEG signals (see properties i and ii in SI Theory, Appendix A.1). For example, coincident non-bursting activity only accounted for about one third of our recordings (average across subjects, connections, and frequencies) so it was not accessible two thirds of the time. One caveat with this abstract approach is that it does not guarantee that the resulting SNR estimates (4) lie above the theoretically minimal value 1 (set by Eq. 8), which would yield ill-conditioned or ill-defined renormalization (see discussion below Eq. 6 in SI Theory, Appendix A.1). This issue turned out to arise in a small fraction of our data, so in practice we conservatively excluded any connection for which the amplitude-specific SNR estimate (13) at any of its two nodes reached below 1.1 in at least one frequency band. This led to the exclusion of no more than 0.45% of our individual functional connectivity dataset (within a single subject) when modeling background noise as non-bursting activity, and 3% (spread over 15 out of the 31 subjects) when referring to measurement noise (i.e., empty-room MEG recordings non-simultaneous to resting-state MEG recordings).

Appendix B.2. More details on statistical procedures

Power bias measure (PBM). We define here explicitly the PBM index in the slightly different cases considered in the main text. Each of them quantifies to what extent the power bias affects amplitude connectivity estimation by assessing the relative difference between the measured value AEC and either the ground truth AEC_0 in simulations or the corrected value $\mathrm{AEC}_{\mathrm{ren}}$ in resting-state data.

For the synthetic functional connectivity data, each of the $N_{\mathrm{subj}} = 30$ "subjects" corresponded to a number of simulations where the true connectivity AEC_0 was varied (simulating in a sense spectral variations of connectivity) while the SNR parameters (4) were kept fixed. The PBM was then taken as

$$\mathrm{PBM} = \frac{\sum_s \left(\langle \mathrm{AEC}^{(s)}\rangle - \langle \mathrm{AEC}_0^{(s)}\rangle\right)^2}{\sum_s \left(\langle \mathrm{AEC}_0^{(s)}\rangle\right)^2}\,. \tag{42}$$

In this section, brackets $\langle\cdot\rangle$ denote group averaging over N_{subj} subjects (rather than time averaging as in SI Theory, Appendix A). Sums run here over all simulations (indexed by superscripts s) performed at fixed SNRs. This PBM index allowed us to quantify the effect of the power bias as a function of the SNR and was used in Fig. 1c; a large PBM indicates a strong effect of the power bias. A closely-related index was used to estimate the power bias correction error

$$\frac{\sum_s \left(\langle \mathrm{AEC}_{\mathrm{ren}}^{(s)}\rangle - \langle \mathrm{AEC}_0^{(s)}\rangle\right)^2}{\sum_s \left(\langle \mathrm{AEC}_0^{(s)}\rangle\right)^2} \tag{43}$$

as a function of the SNR. A large value of this index indicates poor performance of the correction procedure (SI Theory, Appendix A.2, Eq. 15).

For the resting-state MEG connectivity data, the power bias was measured for each connection as

$$\mathrm{PBM} = \frac{\sum_\nu \left(\langle \mathrm{AEC}_{\mathrm{ren}}^{(\nu)}\rangle - \langle \mathrm{AEC}^{(\nu)}\rangle\right)^2}{\sum_\nu \left(\langle \mathrm{AEC}^{(\nu)}\rangle\right)^2}\,, \tag{44}$$

where $\mathrm{AEC}^{(\nu)}$ corresponds to an individual amplitude correlation in the band of center frequency ν. Sums now run over all frequencies ν considered so as to quantify the effect of the power bias on group-averaged AEC spectra. Findings of large PBM demonstrate substantial modifications of connectivity values. This PBM is reported in the main text for interhemispheric network connectivity; a systematic analysis across the whole connectome is developed in SI Results (Appendix C.3). Of note, the square root of the denominator $\sum_\nu(\langle \mathrm{AEC}^{(\nu)}\rangle)^2$ in Eq. (44) corresponds to the "AEC spectrum norm" used to construct Fig. 2c.

Regularized Pearson correlation test of spectral similarities. Parametric correlation testing in power or connectivity spectra requires to know the number n of degrees of freedom in these spectra. We could not estimate it naïvely as the number $N_{\mathrm{freq}} = 45$ of frequency bands because the natural bandwidth of neurophysiological processes leads to a degree of spectral smoothness (i.e., inter-dependent frequencies), so the resulting statistical test would be too lenient. Rather, we regularized our correlation tests in such a way that the null hypothesis adapts to the spectral smoothness, by setting n to an estimate of the number of effectively independent frequencies in the spectra under scrutiny. In practice, we computed the cross-frequency $N_{\mathrm{freq}} \times N_{\mathrm{freq}}$ sample covariance matrix from individual spectra (averaged over the two spectra being correlated) and estimated n as the minimum number of eigenvalues summing up to 99% of the total variance.

Appendix B.3. Statistical procedures for SI Results (Appendix C)

Relative contribution of power bias, noise correlations and linear synchronization. Given that the correction procedure controls for noise correlations and linear synchronization on top of the power bias (see SI Theory, Appendix A.2, Eq. 15), we further assessed the relative impact of the noise correlation term

$$t_{\text{noise}} = \frac{\frac{\text{cov}\left(A_{\epsilon x}^2, A_{\epsilon y}^2\right)}{\sigma_{A_x^2}\sigma_{A_y^2}}}{\sqrt{1-\left(\text{SNR}_x^{\text{ampl}}\right)^{-2}}\sqrt{1-\left(\text{SNR}_y^{\text{ampl}}\right)^{-2}}} \quad (45)$$

and of the synchronization term

$$t_{\text{sync}} = \frac{\frac{\mathscr{L}(x,y)+\mathscr{L}(x,\text{H}y)}{\sigma_{A_x^2}\sigma_{A_y^2}}}{\sqrt{1-\left(\text{SNR}_x^{\text{ampl}}\right)^{-2}}\sqrt{1-\left(\text{SNR}_y^{\text{ampl}}\right)^{-2}}} \quad (46)$$

compared to the main power bias correction term

$$t_{\text{power bias}} = \frac{\text{AEC}}{\sqrt{1-\left(\text{SNR}_x^{\text{ampl}}\right)^{-2}}\sqrt{1-\left(\text{SNR}_y^{\text{ampl}}\right)^{-2}}}\,. \quad (47)$$

Each of these terms were estimated from resting-state MEG data separately for each subject, connection and frequency ν, and the relative contributions of noise correlations and linear synchronization were then measured as the squared spectrum norm ratios

$$\alpha_{\text{noise}} = \frac{\sum_\nu \left\langle t_{\text{noise}}^{(\nu)}\right\rangle^2}{\sum_\nu \left\langle t_{\text{power bias}}^{(\nu)}\right\rangle^2}\,, \quad \alpha_{\text{sync}} = \frac{\sum_\nu \left\langle t_{\text{sync}}^{(\nu)}\right\rangle^2}{\sum_\nu \left\langle t_{\text{power bias}}^{(\nu)}\right\rangle^2}\,. \quad (48)$$

These relative contributions are reported in SI Results (Appendix C.2).

Multiple regression model for power bias correction. The power bias renormalization factor is nonlinear in the SNR (see SI Theory, Appendix A). To examine the importance of this feature, we considered another correction approach based on multiple regression. Regression models are widely used in neuroimaging to discard unwanted effects (covariates of no interest) and highlight effects thought to be relevant (covariates of interest). For each connection and each frequency band of our AEC data, we designed the linear model

$$\text{AEC} = \beta_0 + \beta_x \frac{1}{\text{SNR}_x} + \beta_y \frac{1}{\text{SNR}_y} + \beta_\epsilon R_{\text{noise}} + E \quad (49)$$

and determined the coefficients β_0, β_x, β_y, and β_ϵ in a standard manner by minimizing the group variance of the model error E. To keep the approach generic and independent of the theoretical developments in SI Theory (Appendix A.2), we used the basic definition (4) of the SNR rather than the amplitude-specific form (Eq. 13) identified in the context of the power bias renormalization (Eq. 15), and likewise we used the linear noise correlation R_{noise}. Nevertheless, we set the two SNR-related regressors to the inverse SNR as it is reasonable to expect on general grounds that connectivity estimates AEC converge to their true value AEC_0 in the large SNR limit ($\text{SNR}_x \gg 1$ and $\text{SNR}_y \gg 1$). The true value would then be captured by the intercept regressor corresponding to the first term of the above model. More specifically, β_0 would correspond to the group-average of AEC_0, and the error term E to the inter-individual variations.

The regression-based corrected amplitude connectivity estimate can thus be identified as

$$\text{AEC}_{\text{reg}} = \text{AEC} - \beta_x \frac{1}{\text{SNR}_x} - \beta_y \frac{1}{\text{SNR}_y} - \beta_\epsilon R_{\text{noise}}\,. \quad (50)$$

Again, this was applied independently for each pair x, y of source-projected MEG signals (with one being leakage corrected with respect to the other) and within each frequency band, and symmetrization was imposed afterwards (see Methods in the main text). The adequacy of this approach to power bias correction is examined in Fig. 3 for interhemispheric network connectivity and more systematically in SI Results (Appendix C.3).

Statistical assessment of connectivity changes: regularized Hotelling's T^2 test. The statistical significance of connectivity changes measured by the PBM (Appendix B.2) was established using a version of Hotelling's T^2 test. Specifically, we computed for each connection the squared Mahalanobis distance in dimension $N_{\text{freq}} = 45$ between connectivity spectra $\text{AEC}^{(\nu)}$ before correction and $\text{AEC}_{\text{ren}}^{(\nu)}$ after correction (where ν denotes frequency), under the multivariate null hypothesis $\langle \text{AEC}_{\text{ren}}^{(\nu)} - \text{AEC}^{(\nu)} \rangle = 0$ that the power bias does not affect AEC estimation. One issue in this computation is the non-invertibility of the corresponding $N_{\text{freq}} \times N_{\text{freq}}$ sample covariance matrix due to the inter-dependencies among frequencies (Appendix B.2). To regularize the situation, the covariance was pseudo-inverted using its first n' largest eigenvalues, with n' the number of effectively independent frequencies determined as in the case of the correlation tests (Appendix B.2) but applied here on the sample covariance of the differences between the two spectra being compared. Statistical inference was then performed at significance level $p < 0.05$ using Hotelling's $T^2_{n', N_{\text{subj}}-1}$ distribution in dimension n' with $N_{\text{subj}} - 1$ degrees of freedom. Of notice, N_{subj} was slightly smaller than the total number of subjects (i.e., 31) for a few, sparsely distributed connections due to the technical caveat mentioned in Appendix B.1. The results of this analysis are described in SI Results (Appendix C.4).

Appendix C. SI Results

Appendix C.1. Proof-of-concept of power bias correction in the presence of correlated noises

The inflating effect of noise coupling on AEC estimation (see SI Theory, Appendix A.2, Eq. 12) is illustrated in Fig. S2 using synthetic data that now include various levels of noise amplitude correlation ($\text{AEC}_{\text{noise}}$), but in the absence of neural amplitude coupling ($\text{AEC}_0 \approx 0$). The relationship between AEC and

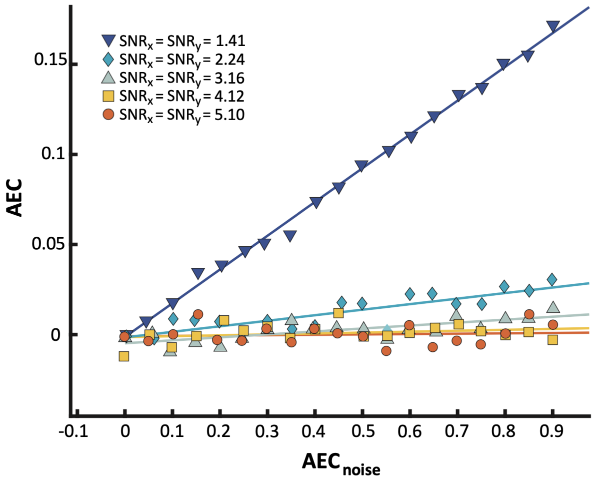

Figure S2: *Linear relationship between AEC estimation and noise amplitude correlation AEC_{noise}. Linear regression curves are superimposed to data points.*

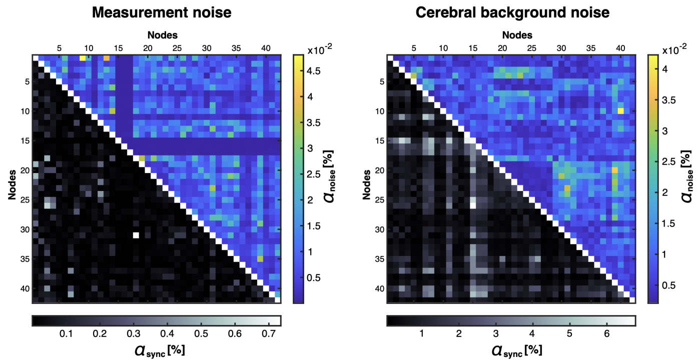

Figure S3: *Relative contribution of correction features.* The impact of noise correlation (α_{noise}) and linear synchronization (α_{sync}) in AEC power bias correction is mapped across the 42-node connectome when using measurement noise (left) and when modeling cerebral background noise as coincident non-bursting activity (right). See SI Methods (Appendix B.3) for precise definitions of these percentages.

AEC_{noise} is linear with a slope that decreased when increasing the SNR, in line with Eq. (12). Noise correlation leads to fairly limited AEC overestimation at SNRs above 2, i.e., when connectivity processes dominate over background noise, but this effect increases substantially as the SNR decreases down to 1.

Even though noise amplitude correlation was subdominant in our resting-state data (see Fig. 2c), we re-assessed the correction errors reported in Fig. 1d by adding noise coupling to the synthetic data, with a fixed level $AEC_{\text{noise}} = 0.2$. Again, simulated neural amplitude coupling was successfully recovered with low correction errors (1% at SNR = 1.4; < 0.1% for SNR > 3). This extends our proof-of-concept in Fig. 1d to the case of correlated noises.

Appendix C.2. Relative contribution of power bias, noise correlations and linear synchronization

Given that our correction method not only controls for the power bias but also for noise correlation and spontaneous linear synchronization (see SI Theory, Appendix A.2, Eq. 15), we assessed the contribution of the two latter factors relative to that of the power bias *per se* (Fig. S3). The contribution of noise correlations was extremely small compared to the power bias itself ($\alpha_{\text{noise}} < 0.05\%$ when measurement noise is used in the correction, see Fig. S3, left, and when cerebral background noise is modeled as non-bursting activity, see Fig. S3, right). This was expected by design of our approach to noise modeling since lack of connectivity was one defining property of noise (see SI Theory, Appendix A.1, property i) and in line with Fig. 2c, although in this case remnant noise correlations might subsist from possible spatial leakage miscorrections (Wens et al., 2015). The contribution of linear synchronization was larger, in line with the possible existence of spontaneous linear synchronization processes (Sjogard et al., 2019), but still highly subdominant compared to the power bias itself ($\alpha_{\text{sync}} < 0.8\%$ when measurement noise is used in the correction, see Fig. S3, left; $\alpha_{\text{sync}} < 7\%$ when cerebral background noise is modeled as non-bursting activity, see Fig. S3, right).

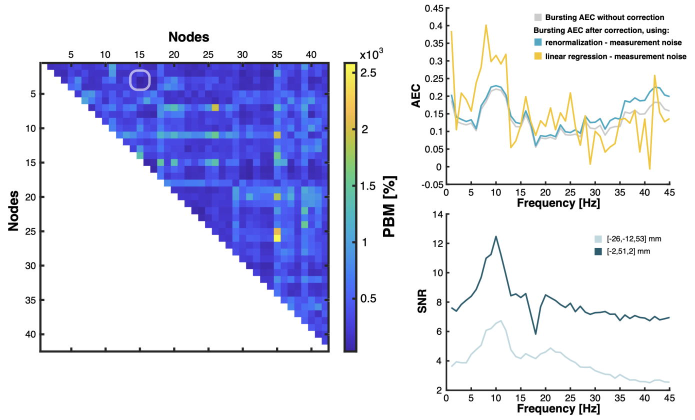

Figure S4: *Power bias correction via linear regression in amplitude correlation spectra of the connectome.* **Left.** The PBM (see SI Methods, Appendix B.2) associated with power bias correction based on regression and measurement noise (see SI Methods, Appendix B.3) was mapped in the whole-brain-covering connectome. **Right.** Functional connectivity (top) and SNR (bottom) spectra of the connection with the least amount of connectivity change. Its location in the connectome is highlighted in the corresponding PBM matrix.

Appendix C.3. Multiple regression model for power bias correction

To assess the importance of SNR nonlinearity in power bias correction, we considered linear regression of node SNRs (along with noise correlation) as an alternative correction approach. For conciseness, we focus here on power bias correction based on measurement noise where our nonlinear renormalization procedure (SI Theory, Appendix A.2, Eq. 15) led to virtually no connectivity changes (see main text and Appendix C.4); the results of regression modeling are thus quickly interpretable in this case.

The example of interhemispheric network connectivity considered in the main text shows that AEC spectra (Fig. 3, top, yellow) corrected by regression are much more erratic than renormalized AEC (Fig. 3, top, blue), especially in the AN. Accordingly, regression led to connectivity changes (SMN, PBM = 110%; AN, PBM = 550%; VN, PBM = 130%) hun-

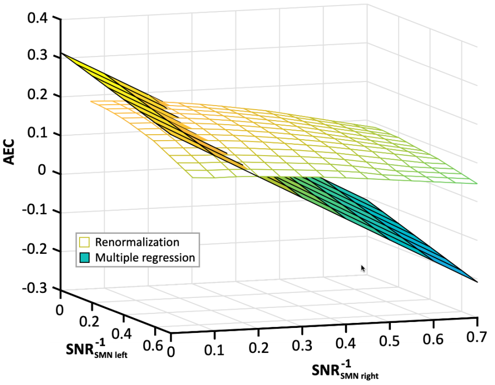

Figure S5: *Comparison of power bias correction and multiple regression, using measurement noise.* This example taken from the resting-state SMN connectivity data at 9 Hz shows two model surfaces used for two distinct correction methods. The planar surface corresponds to multiple regression and the curved one, to power bias correction by AEC renormalization (15). In both cases, the correction consists in extrapolating AEC to the intercept value above $\mathrm{SNR}_x^{-1} = \mathrm{SNR}_y^{-1} = 0$.

dreds of times larger than our renormalization (PBM = 1%, i.e., very small power bias effect size). This suggests that linear regression is inadequate for power bias correction, and that taking into account SNR nonlinearity is fundamental. This observation generalized across the whole connectome (Fig. S4).

We further sought to illustrate the reason for inadequacy of this regression graphically in Fig. S5, where we compare the nonlinear power bias model and the linear regression model from the SMN interhemispheric connectivity data at 9 Hz. The regression surface corresponds to a flat plane whose intercept value (i.e., the linear extrapolation of Eq. 49 in SI Methods, Appendix B.3 to very large SNR values $\mathrm{SNR}_x^{-1} = \mathrm{SNR}_y^{-1} = 0$ where power should not bias functional connectivity) yields the corrected AEC (Eq. 50). By contrast, the nonlinearity of our power bias model (Eq. 12 in SI Theory, Appendix A.2; see also Fig. 1b) yields a curved surface that flattens when approaching the intercept, which led to a lower renormalized AEC (Eq. 15). This nonlinear flattening of the power bias explains why the linear regression tends to overestimate interhemispheric AEC in Fig. 3.

Appendix C.4. Statistical assessment of connectivity changes

In the main text, we focused on the question of whether or not power bias correction modifies the shape of connectivity spectra, not the global level of connectivity. Here we provide a quantitative and statistical analysis of the sheer effect size of connectivity changes brought by the power bias and measured by the PBM index.

Interhemispheric connectivity. When modeling cerebral background noise as non-bursting brain activity, the power bias levelled up interhemispheric network connectivity by about 20% (PBM values; SMN, 18%; AN, 26%; VN, 17%), but these effects were not statistically significant (regularized T^2 test; SMN, $p = 0.065$; AN, $p = 0.27$; VN, $p = 0.051$). The PBM dropped substantially (to 1% for the three networks) when using measurement noise, yet rather counter-intuitively these small effects turned out marginally significant (regularized T^2 test; SMN, $p = 0.007$; AN, $p = 0.053$; VN, $p = 0.025$). This was explained by the observation that our cerebral background noise model based on non-bursting brain states revealed a higher inter-individual variability in corrected functional connectivity (that we presume of biological origin) than measurement noise (which corresponds to highly reproducible empty-room MEG recordings).

Functional connectome. Figure S6 reports a systematic analysis of PBM across the connectome. The strongest effect sizes were located at a connection linking the ventral attention (node MNI coordinates, $[41, 17, 31]$ mm) and the default-mode networks ($[-57, -25, -17]$ mm) with a PBM = 33% change for power bias correction relative to cerebral background noise (Fig. S6, right), and at the already encountered connection linking the dorsal attention ($[-26, -12, 53]$ mm) and the visual networks ($[27, -71, -14]$ mm) with a PBM = 9% change for power bias correction relative to measurement noise (Fig. S6, left). However, none of these connectivity changes turned out statistically significant after controlling for the familywise error rate.

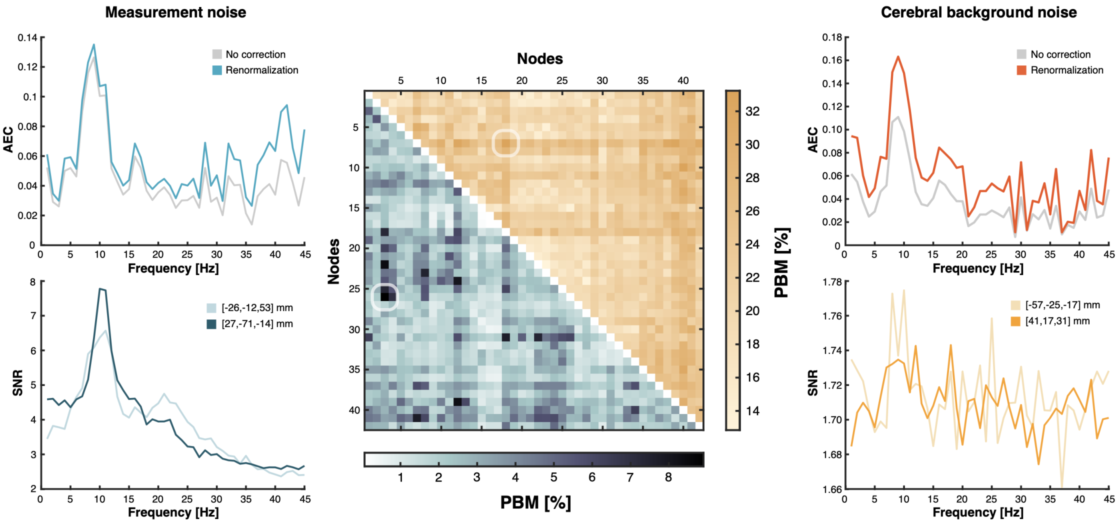

Figure S6: *Power bias effect size in amplitude correlation spectra of the connectome.* **Middle.** The PBM (see SI Methods, Appendix B.2) associated with power bias correction relative to measurement noise (lower-left triangle, blue shades) or to cerebral background noise modeled as non-bursting activity (upper-right triangle, yellow shades) was mapped in the whole-brain-covering connectome. **Left.** Functional connectivity (top) and SNR (bottom) spectra of the connection showing the highest connectivity change when modeling noise as measurement noise. Its location in the connectome is highlighted in the corresponding PBM matrix. **Right.** Same as left, but modeling cerebral background noise as non-bursting brain activity.
